# CDH18 is a fetal epicardial biomarker regulating differentiation towards vascular smooth muscle cells

**DOI:** 10.1038/s41536-022-00207-w

**Published:** 2022-02-02

**Authors:** Julia Junghof, Yuta Kogure, Tian Yu, Eva María Verdugo-Sivianes, Megumi Narita, Antonio Lucena-Cacace, Yoshinori Yoshida

**Affiliations:** 1 Center for iPS Cell Research and Application, Kyoto University, Kyoto, 606-8507 Japan; 2 Graduate School of Medicine, Kyoto University, Kyoto, 606-8501 Japan; 3 Research and Development Division, ROHTO Pharmaceutical Co., Ltd., Osaka, 544-8666 Japan; 4 Instituto de Biomedicina de Sevilla, IBIS, Hospital Universitario Virgen del Rocío, Universidad de Sevilla, Consejo Superior de Investigaciones Científicas, Avda. Manuel Siurot s/n, 41013, Seville, Spain; 5 CIBERONC, Instituto de Salud Carlos III, 28029 Madrid, Spain

**Keywords:** Transcriptomics, Regenerative medicine, Stem-cell differentiation, Cell signalling

## Abstract

The epicardium is a mesothelial layer covering the myocardium serving as a progenitor source during cardiac development. The epicardium reactivates upon cardiac injury supporting cardiac repair and regeneration. Fine-tuned balanced signaling regulates cell plasticity and cell-fate decisions of epicardial-derived cells (EPCDs) via epicardial-to-mesenchymal transition (EMT). However, powerful tools to investigate epicardial function, including markers with pivotal roles in developmental signaling, are still lacking. Here, we recapitulated epicardiogenesis using human induced pluripotent stem cells (hiPSCs) and identified type II classical cadherin CDH18 as a biomarker defining lineage specification in human active epicardium. The loss of CDH18 led to the onset of EMT and specific differentiation towards cardiac smooth muscle cells. Furthermore, *GATA4* regulated epicardial *CDH18* expression. These results highlight the importance of tracing *CDH18* expression in hiPSC-derived epicardial cells, providing a model for investigating epicardial function in human development and disease and enabling new possibilities for regenerative medicine.

## Introduction

Cardiovascular diseases are the leading cause of death worldwide. The human heart is incapable of restoring itself and myocardial repair and regeneration processes are still poorly understood. In recent years the epicardium has emerged as a therapeutic target, given its ability to re-activate upon cardiac injury and promote cardiac repair^1^. The epicardium is a mesothelial layer covering the myocardium and serves as a progenitor source supporting cardiac development, repair, and regeneration^2–4^. Epicardiogenesis is evolutionary conserved and absolutely essential for cardiac development, with failure of epicardial formation being embryonic lethal^5,6^. After the formation of the proepicardium (PE), a transient cauliflower-like structure at the venous pole of the looping heart, cells of the PE migrate over and cover the looping heart tube completely, subsequently forming the epicardium. The embryonic epicardium is active, epicardial cells are proliferative and have the ability to undergo epicardial epithelial-to-mesenchymal transition (EMT). Those epicardial-derived cells (EPDCs) can proliferate and migrate, invading the underlying myocardium, where they subsequently differentiate into various cardiac cell types like vascular smooth muscle cells (SMC), cardiac fibroblasts (CF), and, to a lesser degree, endothelial cells (EC)^7–9^. The epicardium and EPDCs exhibit extensive developmental plasticity, which is crucial for cardiogenesis, by contributing to coronary vessel formation as well as myocardial growth and maturation^1,2,4,10,11^. During adulthood, the epicardium enters quiescence and serves as a protective layer. Only upon cardiac injury, the epicardium re-activates and contributes as a quick transient progenitor hub of derivative cells to the cardiovascular system regeneration^4,12^. This fact provides the epicardium with a pivotal role in mediating regenerative responses. Unlike other species, mammals lack the ability to restore the heart. Nonetheless, re-activation of the epicardium is required for cardiac scar formation as well as coronary vessel growth^13^. Not only promotes the epicardium myocardial regeneration via paracrine signaling, but it also mediates inflammatory responses^14,15^. Moreover, engineered patches carrying epicardial follistatin-like 1 (FSTL-1) were able to enhance cardiac regenerative responses^16^. However, despite ongoing investigation on the epicardium as a therapeutic target tissue, processes governing epicardial development, re-activation and engraftment ability are not well understood, mainly due to lacking comprehension of the fundamental biology behind the epicardium itself, including the expression dynamics of epicardial epitopes mediating tissue responses to homeostasis disruptions and cellular signaling upon cardiac damage.

The epicardium coordinates a complex network of surface remodelers in order to respond to different stimuli driven by organ development and tissue repair^11,17^. The cell surfaceome describes the whole proteome of surface and transmembrane proteins. During tissue morphogenesis, homeostasis, and regeneration, the surfaceome, particularly surface remodelers, plays an essential role in expansion, migration, and invasion, forming complex cellular structures^18^. However, cell-surface markers defining functional epicardial cells or regulating epicardial cell-fate decisions have been difficult to identify.

The cadherin family of cell–cell adhesion proteins are important for tissue morphogenesis^19^. Even though cadherins may have originated to facilitate mechanical cell-cell adhesion, their functions are pleiotropic and have evolved to be imperative for many other aspects, including cell recognition, coordinated cell movements, cell-fate decisions and the maintenance of structural tissue polarity, by controlling diverse signaling pathways^20–25^.

In this study, we identified type II classical cadherin CDH18, formerly known as cadherin 14 (CDH14), as a specific biomarker expressed in the fetal-stage epicardium, defining cellular specification. Human pluripotent stem cells (hPSCs)^26,27^ and their capacity to differentiate into cardiac cells allow recapitulation of epicardiogenesis in vitro^28–36^. Here we generated human-induced (hi)PSC-derived epicardial-like (EPI) cells. Epicardial identity was associated with sustained expression levels of key epicardial genes and retained *CDH18* expression in correlation to *GATA4* expression, an essential transcription factor proven to be required to form PE in vivo^37^. The loss of *CDH18* expression led to the activation of cell-fate specific EMT towards SMC differentiation, confirming an important biological function of *CDH18*, not only to define progressive epicardial lineage specification but also to regulate downstream signaling that drives EPDC derivation. Our study sets a basis for a reproducible model to investigate epicardial function in human cardiac development and disease, ultimately enabling new possibilities in regenerative medicine.

## Results

### Defined transcriptome of developing epicardial surfaceome revealed the enrichment of *CD22* and *CDH18* in late-stage populations

**Fig. 1:**
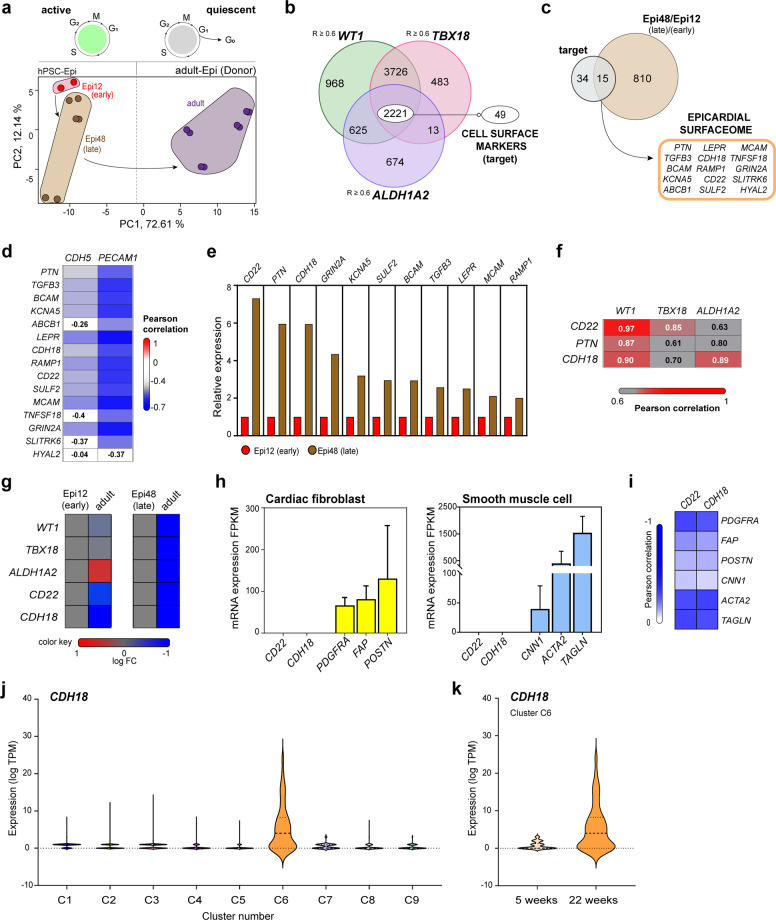
Defined surfaceome transcript of the developing epicardium revealed the enrichment of *CD22* and *CDH18* in late-stage populations. **a** PCA using the GSE84085 RNA-Seq expression dataset of d12 early epicardial-like cells (Epi12, red) (19-9-7-Epi, *n* = 2), d48 late epicardial-like cells (Epi48, brown) (H9-Epi, ES03-Epi and 19-9-11-Epi, *n* = 6) and human adult epicardium (dark violet) (donors 9605, 9633, 9634 and 9635, *n* = 8). **b** Venn-Diagram showing genes in significant absolute correlation to the epicardial markers *WT1* (light green), *TBX18* (light pink) and *ALDH1A2* (light purple) (Pearson correlations; *R* ≥ 0.6) as well as cell surface marker enrichment (target)**. c** Overlap of the target gene set from **b** (gray) against DEGs (Supplementary Fig. 1e) (light brown) for the surfaceome transcript identification of the epicardium. **d** Heatmap showing correlations (Pearson, *R*, 1 to −0.7, color scale) of surface marker expression relative to the expression of endothelial cell markers *CDH5* and *PECAM1*. **e** Fold change of surface marker expression in Epi48 (brown) relative to Epi12 (red). **f** Heatmap showing correlations (Pearson, *R*, 0.6–1, color scale) of the top three genes from (**e**) to epicardial markers. **g** Retrospective analysis showing a heatmap depicting active epicardial markers and *CD22* and *CDH18* expression in embryonic and adult cells. **h** Analysis for *CD22* and *CDH18* expression in CF (left) and SMC (right) with representative CF genes (yellow) and SMC genes (light blue) [error bars indicate standard derivation (SD)]. **i** Heatmap showing correlations (Pearson, *R*, 0 to −1, color scale) of *CD22* and *CDH18* for CF markers *PDGFRA*, *FAP* and *POSTN* and SMC markers *CNN1*, *ACTA2*, and *TAGLN*. **j** GSE106168 retrospective analysis for *CDH18* expression in different cardiac cellular clusters [(C1—5W whole heart (*n* = 257); C2—Cardiomyocytes (*n* = 1492); C3—fibroblast-like cells (*n* = 786); C4—endothelial cells (*n* = 445); C5—heart valves (*n* = 427); C6—epicardial -EPI- cells (*n* = 46); C7—immune cells (*n* = 27); C8—macrophages (*n* = 308); C9—T and B lymphocytes (*n* = 58)] **k** GSE106168 retrospective analysis for *CDH18* expression in epicardial-EPI-cluster [*UPK3B*^high^ *WT1*^high^ *TBX18*^high^ *ALDH1A2*^high^] ranging different time points of gestation [5 weeks, *n* = 30; 22 weeks, *n* = 46].

To investigate the expression profiles of the epicardium, we compared RNA-Seq dataset of induced epicardial-like cells at day (d) 12 (Epi12) and 48 (Epi48) representing a developmental early- and late-stage respectively and investigated their relationship with the adult quiescent epicardium^28^. Whole mRNA transcriptome-based principal-component analysis (PCA) demonstrated 84.75% variance between Epi12 and Epi48 populations (Fig. 1a), with the biggest difference observed between Epi12 and adult—quiescent—cells (Supplementary Fig. 1a, b). The active embryonic epicardium is defined by the expression of the transcription factors Wilms’ tumor 1 (*WT1*) and T-Box 18 (*TBX18*), followed by aldehyde dehydrogenase 1 member A2 (*ALDH1A2*), distinguishing it from the myocardium and endocardium, as well as the adult epicardium which lies quiescent (Supplementary Fig. 1c). All three genes were upregulated in correlation to each other over time (Supplementary Fig. 1d, e), confirming the embryonic epicardial identity of the transcriptome profiles. Comparative analysis of genes in correlation to *WT1, TBX18* and *ALDH1A2*, unveiled 2221 common genes in their intersection (Fig. 1b), with 49 genes identified as surface protein-encoding. We also found 825 differentially expressed genes (DEGs) between Epi12 and Epi48 populations (Supplementary Fig. 1f) allowing us to subsequently define a putative transcriptome of epitopes of the active epicardium (Fig. 1c).

Next, we excluded genes that had no significant negative correlation to the endothelial cell markers *CDH5* and *PECAM1* (Fig. 1d) and selected late-stage upregulated genes (Fig. 1e) that showed a highly positive correlation to epicardial genes (Fig. 1f), obtaining *CD22* and *CDH18* as candidate cell surface biomarkers in the active epicardium. We then confirmed that both candidates were no longer expressed in adult tissue similar to active epicardial key genes (Fig. 1g and Supplementary Fig. 1g). Next, we verified their absence in two important epicardial derivatives. While CF and SMC markers were upregulated in their respective cellular identities, no mRNA expression of *CD22* or *CDH18* was detected (Fig. 1h, i). Moreover, we confirmed tissue specificity using independent datasets: Heart *CD22* expression ranked #2 after central nervous system (CNS), and heart *CDH18* expression ranked #3 after CNS and testis (Supplementary Fig. 1h), two known CDH18-expressing tissues^38–40^. Finally, as an increase of CDH18 is difficult to observe during heart development of mouse embryos due to cellular heterogeneity in the bulk population (Supplementary Fig. 1i), we organotypically cultured explanted epicardial tissue from E14 hearts and documented CDH18 expression in epicardial explants (Supplementary Fig. 1i). We confirmed the epicardial specificity of CDH18 expression in a physiological context of cardiac cells derived from human embryos ranging from different time points of gestation (Fig. 1j, k).

### CDH18 but not CD22 is an epicardial biomarker defining progressive lineage specification

**Fig. 2:**
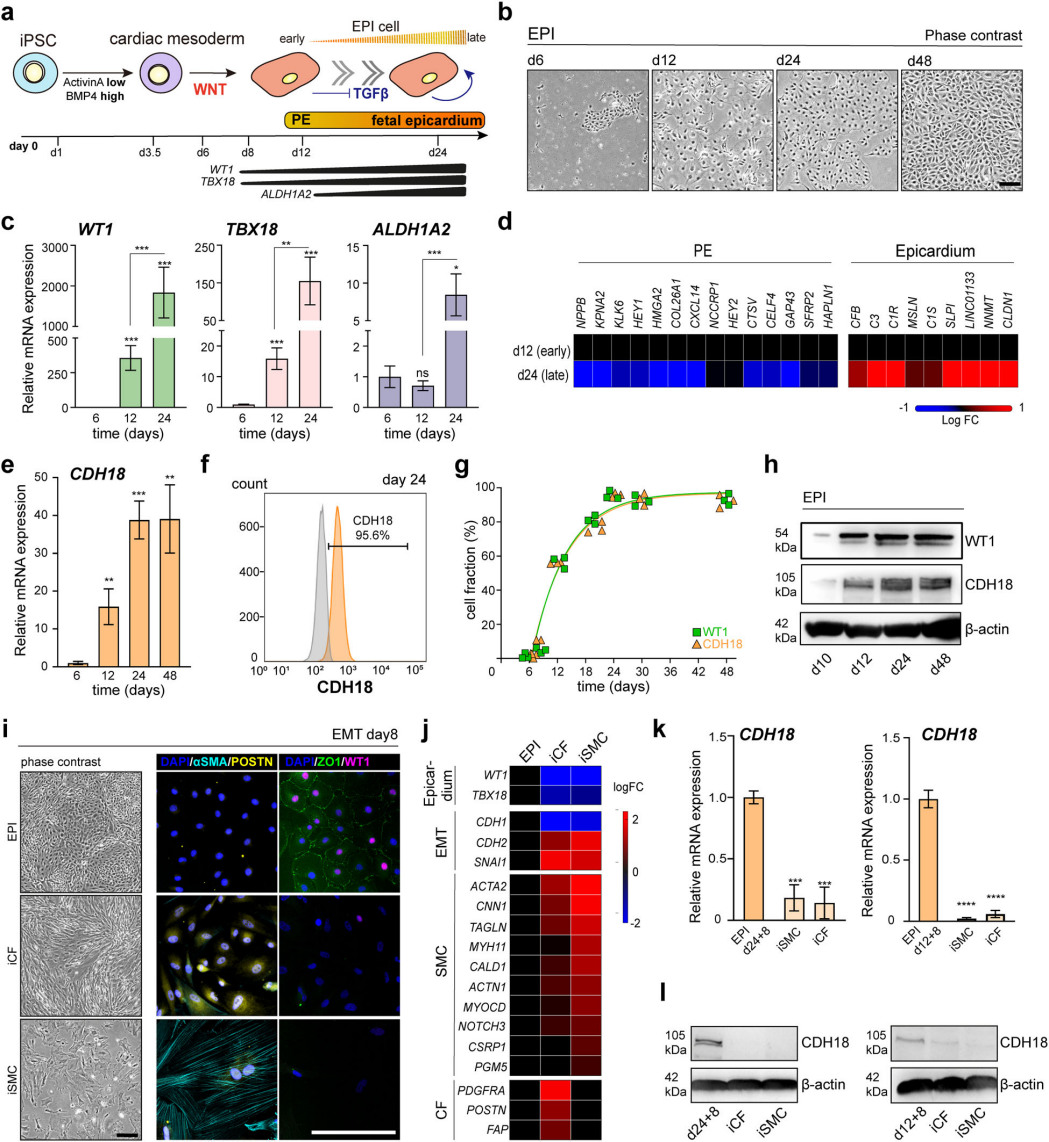
CDH18 but not CD22 is an epicardial biomarker defining progressive lineage specification. **a** Schematic illustration depicting the epicardial differentiation from hiPSCs and representative developmental stages. **b** Phase contrast microscopy showing the morphology of EPI cells from d6-48. **c** qRT-PCR analysis of *WT1* (light green), *TBX18* (light red) and *ALDH1A2* (light purple) during induction normalized to d6. [d6 *n* = 7, d12 *n* = 10, d24 *n* = 10; *p < 0.05, **p < 0.005, ***p < 0.0005]. **d** Heatmap showing PE and primitive epicardium gene signature enriched in d12 and d24 EPI cells respectively. **e** Time course of *CDH18* expression levels (orange) normalized to d6 [d6 *n* = 4, d12 *n* = 4, d24 *n* = 8, d48 *n* = 8; **p < 0.01, ***p = 0.0008]. **f** Flow cytometry analysis for CDH18 (orange) [gray, unstained control]. **g**, Nonlinear-regression fit curve depicting WT1 (green, squares) and CDH18 (orange, triangles) co-expression in EPI cells [*n* = 3]. **h** Western blot analysis showing the co-expression of WT1 and CDH18 in EPI cells during induction. **i** Immunocytochemistry of the EPDC markers POSTN (CF) and αSMA (SMC) and the EPI markers ZO1 and WT1 for EMT validation [fluorescence microscopy images were pseudo-colored using BZ-X analyzer software]. **j** Heatmap of epicardium, EMT, SMC and CF gene signatures [GSE165450]. **k**, **l** *CDH18* expression analyzed by **k** qRT-PCR in induced derivatives (light orange) from d24 cells (left) and d12 cells (right), normalized to the respective EPI control cells (orange) [*n* = 3; one-way ANOVA with Dunnett’s multiple comparison test, ***p < 0.0005] with **l** corresponding western blot analysis. [statistical analysis performed by Mann–Whitney-test, unless otherwise indicated; all error bars represent standard error of the mean (SEM); scale bars 100 µm].

To define the expression pattern of *CD22* and *CDH18*, we first recapitulated epicardiogenesis in vitro. We modified previously reported induction methods for hiPSC-derived epicardial-like (EPI) cells^28,32,35^ to robustly yield WT1^+^ cells at a higher rate, and long-term culture of cells was achieved by permanent inhibition of transforming growth factor (TGF)-β (Fig. 2a). As epicardial cells and atrial cardiomyocytes share a common progenitor pool during development^3^, we excluded the presence of any residual myocyte traces in our system, by generating EPI cells from MYH6 reporter line (MYH6-EIP4)^41^, obtaining no cardiomyocytes (Supplementary Fig. 2a–c). Moreover, the absence of endothelial markers CD31 and CD144 ensured that no cells of endothelial nature were present (Supplementary Fig. 2d–f). EPI cells showed typical epicardial cobblestone-like morphology (Fig. 2b), and key active epicardial genes (*WT1* and *TBX18)* started to be upregulated as early as d12 continuing so until d24 (Fig. 2c). In agreement with previous models, cells did not upregulate *ALDH1A2*—a gene highly expressed in the epicardium but not PE—until d24, indicating that the cells at d12 represented an early stage during development comparable to PE cells and specified into later stage epicardium at d24 (fetal-like) (Fig. 2c, d). We found all genes expressed in correlation to each other (Supplementary Fig. 3a) and did not observe significant differences in their expressions after d24 (Supplementary Fig. 3b, c), endorsing that cells had completed differentiation into fetal-stage epicardium by d24. We also confirmed WT1 protein expression on this day by immunofluorescence (Supplementary Fig. 3d–f).

We then checked *CD22* expression levels during epicardial differentiation. We found *CD22* expression increased but failed to detect CD22 protein in d24 EPI cells, suggesting protein translation insufficiency, and therefore invalidating CD22 as a suitable biomarker (Supplementary Fig. 4).

Next, we investigated *CDH18* levels and could verify its expression in EPI cells derived from different hiPSC lines (Fig. 2f and Supplementary Fig. 5a). Both mRNA and protein (Fig. 2e–g) levels increased exponentially over time of epicardial differentiation and CDH18 was co-expressed with WT1 in a highly correlated fashion (Fig. 2g, h and Supplementary Fig. 5), implying a potential role during epicardial specification.

Epicardial cells have the potential to transit into EPDCs via EMT and subsequently differentiate into CF and SMC during development and also after re-activation upon cardiac injury. This functionality has also been well reported in vitro by treatment with basic fibroblast growth factor (bFGF) and TGF-β^29,31,34^. We initiated an EMT response (Supplementary Fig. 6a) and observed changed morphology as well as loss of WT1 and ZO1 indicating loss of epicardial identity and demonstrating the functionality to transition into EPDCs (Fig. 2i). The identity of induced CFs (iCFs) and induced SMCs (iSMCs) was validated by expression of POSTN and αSMA respectively (Fig. 2i). We also sequenced the whole transcriptome of iCFs and iSMCs, verifying distinct cellular identities by PCA (Supplementary Fig. 6b), as well as enrichment of respective gene signatures (Fig. 2j). We then analyzed *CDH18* expression in epicardial derivatives from both PE-like d12 and fetal-like d24 EPI cells. *CDH18* was lost in iCF and iSMC (Fig. 2k, l and Supplementary Fig. 6c–f), demonstrating that reduced levels of *CDH18* correlated to a loss of EPI identity. Loss of CDH18 upon EMT was also confirmed in murine epicardial MEC1 cell line (Supplementary Fig. 6d).

Taken together, our results demonstrate that CDH18 is a specific active epicardial biomarker and suggest a causal role in specification and maintenance of epicardial identity.

### Loss of *CDH18* triggers cell-fate specific EMT towards SMCs

**Fig. 3:**
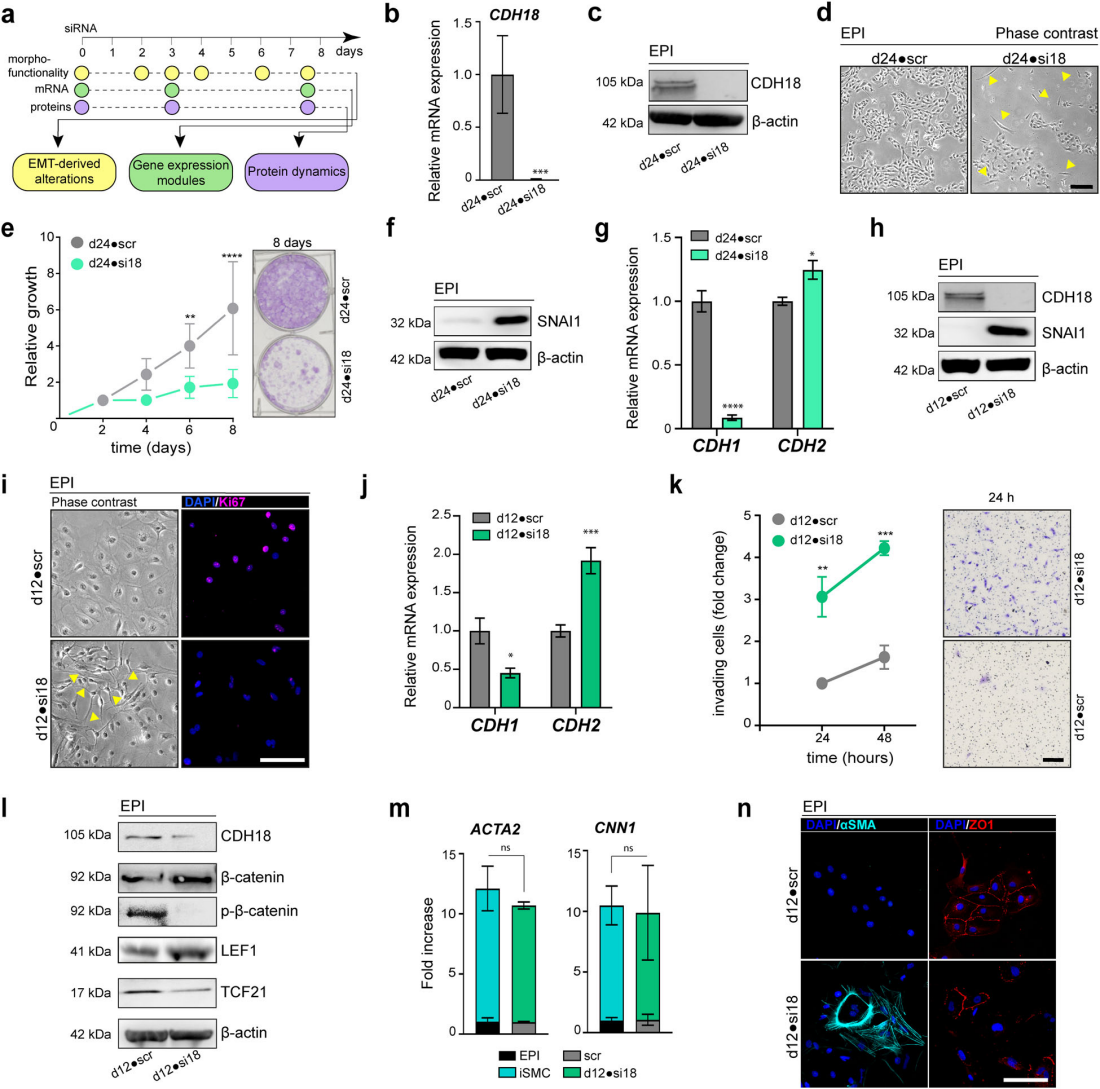
Loss of *CDH18* triggers cell-fate specific EMT towards smooth muscle cells. **a** Illustration of the experimental workflow. **b** and **c** Downregulation of **b** *CDH18* [*n* = 3; unpaired Students *t*-test, ***p = 0.0003] and **c** CDH18 protein expression after 8 days of *CDH18* silencing in d24 EPI cells (d24•si18). **d** Phase contrast microscopy displaying d24•si18 cells with lost cobblestone morphology and elongated shape (yellow arrowheads). **e** Growth curve of d24 cells silenced for *CDH18* (green) and control (scr) (gray) for 2-8 days [*n* = 3; **p = 0.0038, ****p < 0.0001]. **f** SNAI1 western blot of d24•si18 cells. **g** *CDH1* and *CDH2* expression in d24•si18 cells [control (scr); *n* = 3; *p = 0.0141, ****p < 0.0001]. **h** Western blot of CDH18 and SNAI1 after 8 days of *CDH18* silencing in d24 EPI cells (d12•si18). **i** Microscopy analysis of d12•si18 showing changed morphology (yellow arrowheads) and loss of Ki67 (pink) expression [scale bar 50 µm]. **j** Expression of *CDH1* and *CDH2* in d12•si18 EPI cells compared to their control cells [*n* = 3; *p = 0.0231, ***p = 0.0001]. **k** Boyden chamber assay evaluating cell migration capacity in d12•si18 cells [*n* = 3; **p = 0.002, ***p = 0.0004]. Right panel shows invaded cells stained by crystal violet. **l** Western blot analysis showing reduction of CDH18 and TCF21, increased LEF1 and β-catenin with decreased phosphorylated (p)-β-catenin. **m** Expression analysis of SMC markers *ACTA2* and *CNN1* in d12•si18 cells and iSMC [*n* = 3; unpaired Students *t*-test: ns = not significant]. **n** Immunocytochemistry analysis of αSMA and ZO1 expression in d12•si18 cells [scale bar 50 µm]. [statistical analysis performed by two-way ANOVA with Sidak’s multiple comparison test, unless otherwise indicated; all error bars represent SEM; scale bars 100 µm, unless otherwise indicated].

To explore the biological relevance of *CDH18*, we next silenced *CDH18* gene expression in d24 EPI cells (d24•si18) (Fig. 3a). The downregulation of *CDH18* (Fig. 3b–d) led to visible morphological changes with a fraction of cells becoming elongated and losing the cobblestone-like morphology (Fig. 3d, yellow arrowheads) indicating a switch to mesenchymal cells^42^. Cells that retained epicardial morphology were in close contact, suggesting that cell–cell interactions might play a role in maintaining EPI identity. Similar results were obtained using a second siRNA (Supplementary Fig. 7a–c). *CDH18-*silenced cells showed reduced growth (Fig. 3e) suggesting that *CDH18* downregulation in fetal-stage epicardium may confer either a proliferative disadvantage impairing epicardium homeostasis and expansion or define the triggering signal for EMT initiation. We observed a gain of SNAI1 expression (Fig. 3f) upon *CDH18* downregulation, thus indicating the initiation of EMT. Confirming this functional onset, we observed a strong downregulation of *CDH1* (E-cadherin). However, upregulation of *CDH2* (N-cadherin) (Fig. 3g) was only modest.

A previous report showed that epicardial cells are more prone to undergo EMT during the early stages of development^43,44^. We, therefore, questioned, if fetal-like d24 EPI cells were more resistant to initiate EMT than d12 EPI cells, a time-point that portrays a more PE-like state. Accordingly, we silenced *CDH18* in d12 EPI cells (d12•si18) finding similar morphofunctional changes as in d24 EPI cells (Fig. 3h–j and Supplementary Fig. 7d–f). Loss of CDH18 was accompanied by gain of SNAI1 (Fig. 3h) and d12•si18 cells showed changed morphology, reduced growth as well as decreased levels of Ki67 (Supplementary Fig. 7f, g and Fig. 3I), thus indicating the onset of EMT. Notably, the switch from *CDH1* to *CDH2* was much stronger in d12 silenced cells, implying the acquisition of mesenchymal identity (Fig. 3j). Furthermore, *CDH18*-silenced cells showed enhanced migration (Fig. 3k), endorsing that the downregulation of *CDH18* leads to a loss of epicardial identity towards the EPDC-like state. To exclude that our observations were biased due to an early loss of cells we also confirmed that cells displayed reduced proliferation marker expression Ki67, upregulation of SNAI1 and loss of ZO1 3–4 days after silencing (Supplementary Fig. 7h–o), further strengthening, that cells were undergoing EMT upon CDH18 downregulation.

Several pathways are reported to be involved in the regulation of EMT^45–54^, with Wnt pathway regulation by Wt1 being linked to epicardial development and cell differentiation^6^, as well as cardiac regeneration^55^. Cadherins are known to exert their functions through phosphorylation-mediated protein stabilization of β-catenin in the Wnt signaling pathway^56,57^. Moreover, a previous report has shown the direct interaction of Cdh18 with β-catenin^58^. We, therefore, hypothesized that CDH18 might manage its impact on epicardial EMT via the regulation of β-catenin. Indeed, we observed that loss of CDH18 leads to increased levels of β-catenin with decreased phosphorylated (p)-β-catenin as well as an increase in LEF1 (Fig. 3l and Supplementary Fig. 7m, n), indicating the activation of the Wnt pathway.

Interestingly, we found reduced levels of TCF21 upon *CDH18*-silencing (Fig. 3l). *TCF21* acts as an epicardial marker playing pivotal roles during EMT and EPDC-fate regulation between CF and SMC^59–61^. EPI cells showed increased *TCF21* expression at the mRNA and protein levels (Supplementary Fig. 8a, b). Reduced levels of TCF21 imply differentiation towards an SMC rather than a CF fate^7,60^. To confirm this hypothesis, we compared the expression levels of SMC genes in *CDH18*-silenced EPI cells and iSMCs. We found that *CDH18*-silenced cells recapitulated the iSMC expression levels of *ACTA2* and *CNN1* especially in d12 silenced cells and to a lesser degree also in d24-silenced cells (Fig. 3m and Supplementary Fig. 8c–e). Canonical TGFβ-pathway activation seemed to play a noteworthier role for EMT onset in fetal-like d24 compared to PE-like d12 cells, as TGFβ inhibitor withdrawal does enhance the expression levels of *ACTA2* in fetal-like d24 cells (Supplementary Fig. 8e). To assess the role of non-canonical activation, we tested the impact of ROCK, inhibitor impairing RhoA-mediated pathway, on CDH18 downregulation in d12 cells. While no significant change could be observed in SNAI1 expression, *ACTA2* expression was reduced by ROCK inhibition, albeit only when TGFβ signaling was not blocked (Supplementary Fig. 8f). The acquisition of an SMC-like identity in *CDH18*-silenced cells was further verified by the loss of ZO1 and the expression of α-SMA (Fig. 3n).

**Fig. 4:**
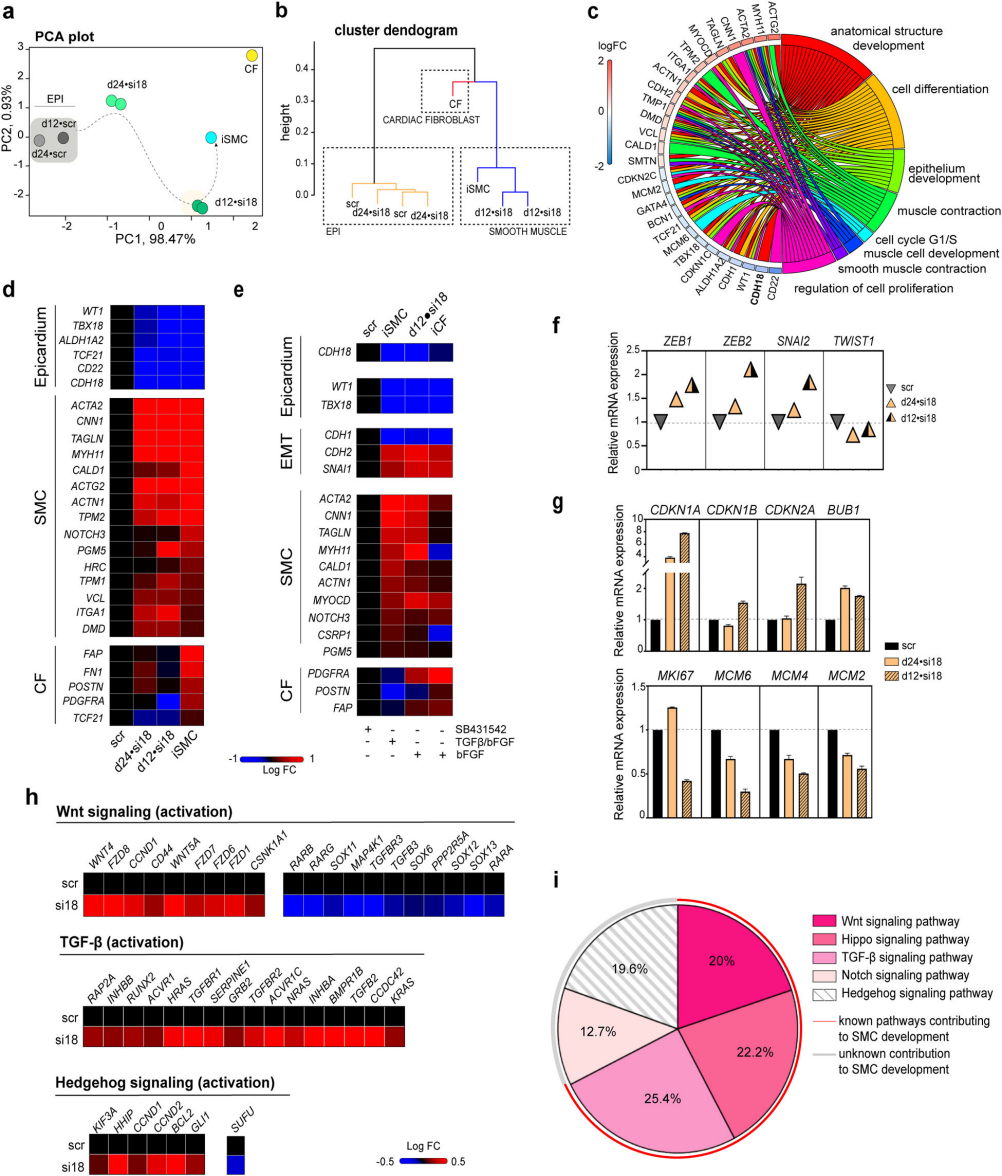
Transcriptional profiling of *CDH18*-silenced EPI cells. **a** PCA of *CDH18*-silenced d12 and d24 EPI cells as well as induced EPDCs compared to unspecific siRNA-treated control (scr) [d24•si18, *n* = 2; d12•si18, *n* = 2; iSMC, *n* = 1; CF; *n* = 1]. **b** Agglomerative hierarchical sample cluster: The Agnes dendrogram was plotted by applying Manhattan distances between samples. **c** Chord diagram representing the flow and the detailed relationship between d12•si18 DEGs (left semicircle perimeter) and their enriched GO biological processes (right semicircle perimeter). **d** Heatmap showing the expression analysis of the gene set enriched for epicardial, SMC and CF genes in iSMCs, *CDH18*-downregulated and control (scr) cells. **e** Heatmap showing the expression analysis of the gene set enriched for epicardial, SMC and CF genes in *CDH18*-downregulated and control (scr) cells under different culture conditions. **f**, **g** Expression levels for **f** EMT specific transcription factors (EMT-TFs) *ZEB1*, *ZEB2*, *SNAI2* and *TWIST1* and **g** *CDKN1A*, *CDKN1B*, *CDKN2A*, *BUB1*, *MKI67*, *MCM6*, *MCM4* and *MCM2* in *CDH18*-downregulated cells. **h** IPA analysis of *CDH18*-silenced d12 EPI cells (si18) versus control (scr) cells showing the activation pattern of signature genes involved in major important developmental pathways. **i** Pathway enrichment analysis showing the proportional correlation of genes affected in important developmental pathways contributing to SMC differentiation. [analysis of Fig. 4 was based on RNA-Seq dataset GSE165450].

Next, we aimed to investigate the transcriptional contribution of *CDH18*-downregulation to the SMC phenotype and the enhanced potential to derive SMC-like cells from d12 PE-like cells compared to d24 fetal-like cells. We, therefore, performed RNA-Seq and showed a transcriptome distribution of up to 99.4% combined variance by PCA (Fig. 4a). PE-like *CDH18*-silenced cells were allocated within the same arm as iSMCs, mapped closer to iSMC and mapped more DEGs (Fig. 4a, b and Supplementary Fig. 9a, b). The downregulation of *CDH18* suppressed epicardial identity both at the fetal-like stage and PE-like stage and activated the SMC gene program, while CF-related genes remained unaffected (Fig. 4c, d and Supplementary Fig. 9c). Specifically, in d12 PE-like stage cells silencing of *CDH18* influenced gene categories supporting SMC acquisition (Fig. 4c) and showed a greater expression of important markers of EMT activation (Fig. 4f). The altered expression of important cell cycle checkpoint regulators confirmed reduced proliferation upon *CDH18* downregulation (Fig. 4g). Finally, we analyzed gene signatures to identify activated signaling pathways, finding that TGF-β and Wnt pathways, which are known to contribute to the SMC phenotype, were affected and activated upon *CDH18* downregulation (Fig. 4h, i).

Altogether, we confirmed that *CDH18* is an important regulator of the SMC phenotype. *CDH18* downregulation led to a loss of epicardial cell identity under the retained inhibition of TGF-β, a situation that normally sustains epicardial hallmarks. The role of *CDH18* is essential in epicardial fate and plasticity determination during human development, especially if the loss of *CDH18* occurs at an early stage of epicardial specification.

### *CDH18* is unable to block TGF-β-driven EMT towards SMC differentiation

**Fig. 5:**
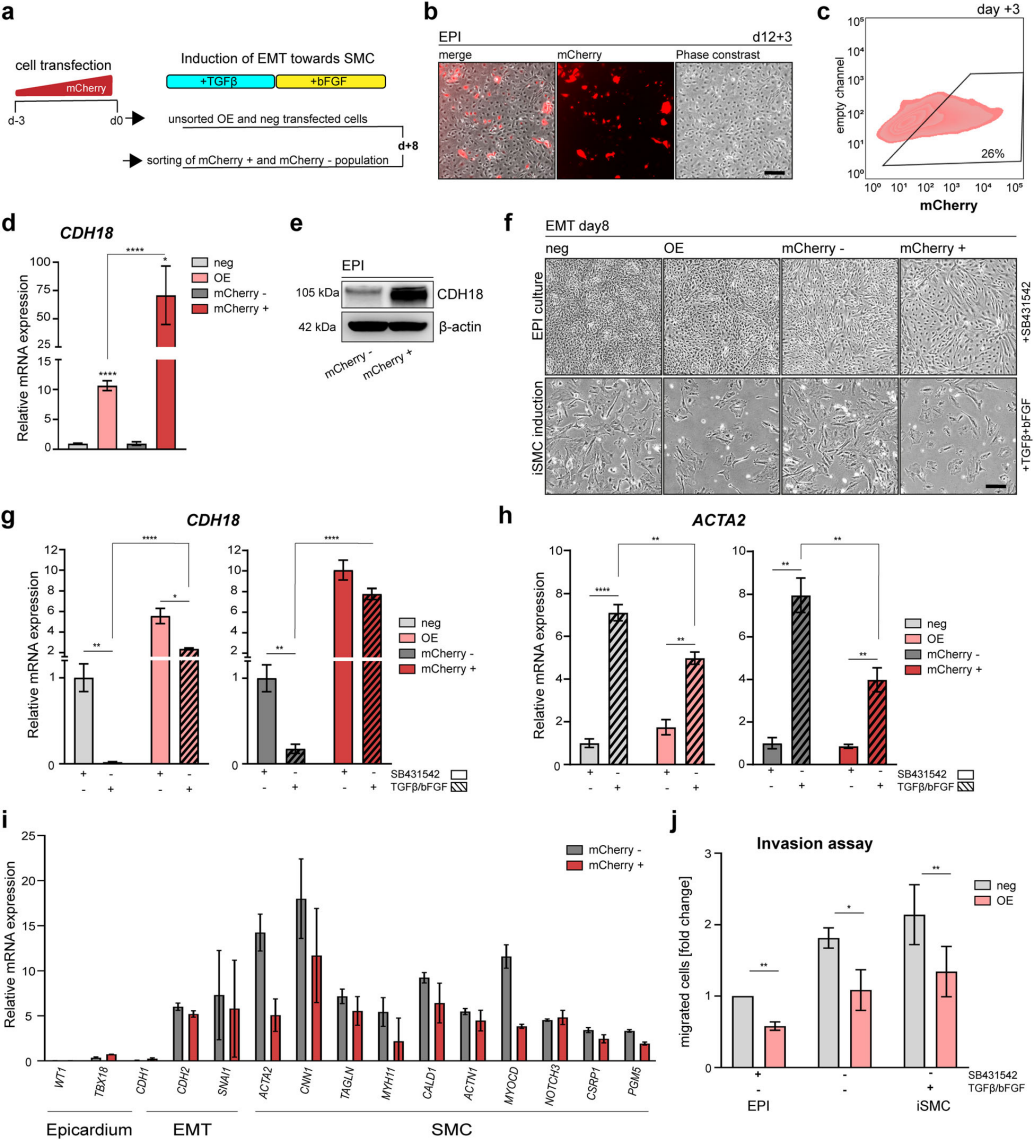
*CDH18* is unable to block TGF-β-driven EMT towards SMC differentiation. **a** Schematic illustration depicting the experimental workflow. **b** Fluorescence microscopy pictures showing mCherry-expressing cells 3 days’ post transfection (dpt) of d12 EPI cells. **c** Flow cytometry analysis showing mCherry expression in EPI cells overexpressing *CDH18* 3 dpt [gating set using empty-transfected cells (Supplementary Fig. 5b)]. **d**, **e** Validation of ectopic *CDH18* cDNA overexpression demonstrating the increase of **d** *CDH18* by qRT-PCR and **e** CDH18 protein by western blot analysis upon plasmid transfection and sorting. **f** Phase contrast pictures of un-/sorted *CDH18*-overexpressing cells induced towards iSMC (+TGF-β + bFGF) or cultured in EPI maintaining conditions (+SB4321542). **g**, **h** Expression of **g** *CDH18* and **h** *ACTA2* in un-/sorted *CDH18*-overexpressing cells and control cells under both EPI maintaining (+SB4321542) and iSMC inducing (+TGF-β + bFGF) culture conditions. **i** Expression analysis based on RNA-Seq dataset [GSE165450] of selected SMC marker genes. **j** Boyden chamber assay evaluating cell migration capacity in d12•si18 cells [*n* = 3; paired Students *t*-test; *p = 0.0336, **p < 0.0054]. [unsorted transfected cells (OE), empty-transfected cells (neg), sorted mCherry-expressing cells (mCherry+), sorted non-mCherry-expressing cells (mCherry-); statistical analysis performed by Repeated measure (RM) one-way ANOVA unless indicated otherwise: ****p < 0.0001, ***p < 0.0005, **p < 0.005, *p < 0.05; all error bars represent SEM; scale bars 100 µm].

To investigate the potential of *CDH18* expression to regulate epicardial maintenance and SMC differentiation, we evaluated the capacity of *CDH18* to inhibit the initiation of EMT towards SMC differentiation. We ectopically overexpressed human *CDH18* cDNA (Supplementary Fig. 10a) in d12 EPI cells, which we assessed via mCherry expression, and subsequently induced EMT towards iSMC formation (Fig. 5a). Unfortunately, EPI cells were difficult to transfect, consistent with a previous report on mouse epicardial-like cells^34,48^. Optimization of the transfection efficiency (Supplementary Table 1) led to ~25% of cells expressing mCherry at d3 post-transfection (Fig. 5b, c and Supplementary Fig. 10b). To obtain a pure *CDH18*-overexpressing population, we then sorted mCherry-expressing cells (mCherry+) (Supplementary Fig. 10c). The gain of *CDH18* expression was confirmed in unsorted and sorted settings (Fig. 5d), and CDH18 protein gain was confirmed in mCherry+ cells (Fig. 5e).

Next, we analyzed the effect of *CDH18* overexpression during iSMC induction. We did not observe morphological differences between conditions (Fig. 5f). Nevertheless, *CDH18*-overexpressing cells showed fewer iSMCs (Fig. 5f), hinting at a potential role of *CDH18* in the maintenance of the active epicardium. All populations showed less *CDH18* expression upon treatment with TGF-β and bFGF (Fig. 5g), but transfected cells retained *CDH18* expression even after undergoing EMT. In agreement with the morphological SMC phenotype (Fig. 5f), all populations showed increased *ACTA2* expression upon iSMC induction (Fig. 5h). Notably, *ACTA2* levels were significantly reduced in cells still expressing *CDH18*. RNA-Seq analysis revealed the reduced expression of several SMC markers in CDH18-overexpressing SMCs (Fig. 5i), highlighting an important role of CDH18 in preventing the fully SMC identity acquisition. During development, not only the differentiation of epicardial cells into SMC, but also their migration is important for successful coronary artery formation^62–64^. To investigate the functional properties of *CDH18*-overexpressing cells, we next tested their invasion ability. We compared EPI cells to cells undergoing spontaneous EMT in absence of TGF-β inhibitor and CDH18-overexpressing SMCs (Fig. 5j). In all conditions, *CDH18*-overexpressing cells showed reduced invasion capacity. We, therefore, concluded, that although ectopic *CDH18* overexpression cannot fully block the TGF-β-driven differentiation to SMC, it partially impairs the acquisition of SMC identity and their functional properties.

### *CDH18* expression is under the control of *GATA4*

**Fig. 6:**
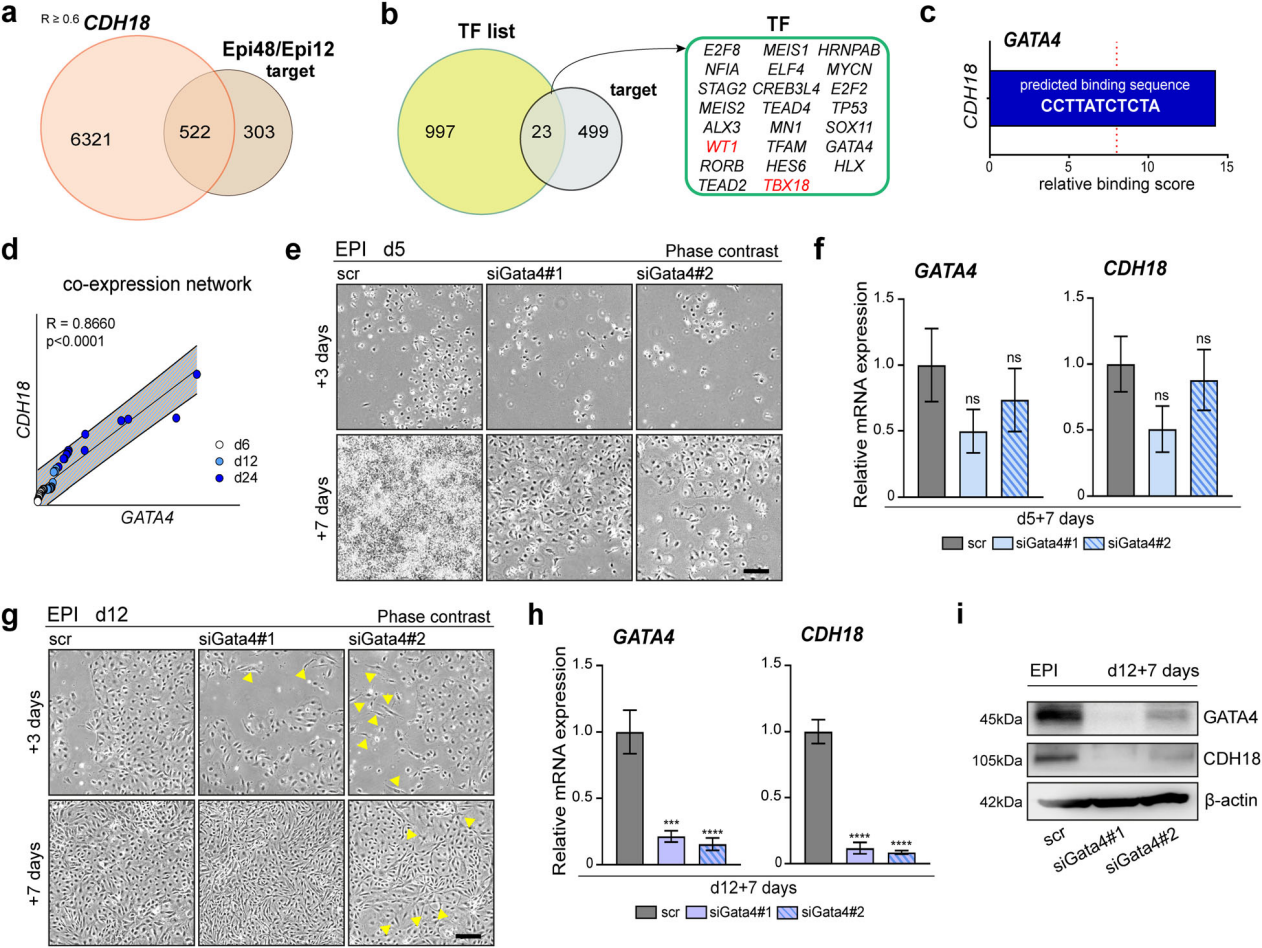
*CDH18* expression is under the control of *GATA4*. **a** Venn diagram depicting the interface of positively correlative genes to *CDH18* (R ≥ 0.6) (light orange) and DEGs between early (Epi12) and late (Epi48) stage epicardium (target) (light brown) [GSE84085] revealed a total of 522 genes within their intersection. **b** Classification of 23 transcription factors (TFs) by overlapping the list of TFs (bright green) and genes identified in **a** (gray). **c** Binding site analysis showing the predicted binding sequence of *GATA4* and relative binding score. **d** Co-expression network analysis of the qRT-PCR data of *GATA4* (Supplementary Fig. 6c) and *CDH18* (Fig. 2f) expression in EPI cells over time. **e**, **f** Silencing of *GATA4* in d5 cells using two different siRNAs (siGata4#1 and siGata4#2) [*n* = 3] show **e** a reduction in epicardial cell density after 3 and 7 days and **f** the expression levels of *GATA4* and *CDH18* after 7 days [ns = not significant]. **g**–**i** Silencing of *GATA4* in d12 cells using two different siRNAs (siGata4#1 and siGata4#2) [*n* = 3] shows **g** morphological changes (blue arrowheads) after 3 and 7 days and **h** reduction of *GATA4* and *CDH18* expression, as revealed by qRT-PCR [***p < 0.0005, ****p < 0.0001], as well as **i** loss of GATA4 and CDH18 protein expression, as shown by western blot analysis. [statistical analysis performed by one-way ANOVA with Dunnett’s multiple comparison test, unless indicated otherwise; all error bars represent SEM; scale bars 100 µm].

Finally, to elucidate the regulation of *CDH18* in epicardiogenesis, we identified 23 transcription factors (TFs) out of 522 genes that are in correlation with *CDH18* and enriched in the late-stage active epicardium (Fig. 6a, b). Among these TF encoding genes, only GATA4 showed a high relative score to bind the *CDH18* promoter (Fig. 6c and Supplementary Fig. 11a). During EPI induction *GATA4* is expressed in associative correlation to *CDH18* (Supplementary Fig. 11b, c and Fig. 6d). *GATA4* is a well-characterized regulator at early cardiogenesis playing a pivotal role in PE formation. *GATA4*-null mice embryos fail to form the epicardium due to impaired PE development, thus being an essential gene in epicardiogenesis^37^. We, therefore, silenced *GATA4* using two siRNAs (siGata4#1 and siGata4#2) at different time points during epicardial differentiation: at d5 of epicardial induction marking the beginning of cell development towards PE-like stage and at d12 marking the specification towards fetal-like epicardium (Supplementary Fig. 11d). *GATA4*-silenced cells showed reduced cell survival (Fig. 6e, upper panel), but surviving clones did not alter *GATA4* levels (Supplementary Fig. 11e) and were able to reconstitute after 7 days (Fig. 6e, lower panel) showing no significant reduction of either *GATA4* or *CDH18* expression (Fig. 6e, f). Thus, implying that upon efficient silencing of *GATA4*, cells were not able to survive. We, therefore, concluded that *GATA4* is necessary for cells at d5 to differentiate towards the PE-like state. Next, we downregulated *GATA4* in d12 EPI cells and observed some morphological changes after siRNA administration over time (Fig. 6g, yellow arrowheads). The downregulation of *GATA4* was accompanied by *CDH18* downregulation in a similar fashion on both mRNA (Fig. 6h and Supplementary Fig. 11f) and protein level (Fig. 6i). Thus, these results promote *GATA4* as a putative regulator of *CDH18* expression, highlighting their relevance for the formation and specification of the active epicardium.

## Discussion

The epicardium is essential for cardiogenesis and its re-activation upon injury during adulthood is indispensable for cardiac regeneration. Exploiting epicardial targeting to model cell therapy-based approaches for cardiac repair and regeneration requires detailed knowledge of the mechanisms that regulate the active epicardium. Here, we recapitulated the human epicardiogenesis using hiPSCs and defined a surfaceome of human active epicardium that is later inactivated in the quiescent (adult) epicardium. We define *CDH18* as an epicardial biomarker that is exclusively expressed in epicardial cells compared to their derivatives and other cardiac cell types. The epicardium specificity of *CDH18* expression can be used as a specific, rather than associated, active epicardial biomarker to both define biological identity and modulate EMT-specific processes towards SMC. Previous identification of epicardial cells was based on the expression of several transcription factors such as *WT1*, *TBX18* and *TCF21*. However, their expressions are not restrictive to the epicardium. Our findings provide a molecular alternative for the identification of epicardial cells by tracing *CDH18* expression, a biomarker of the active fetal epicardium.

Cadherin 18 (CDH18), formerly named cadherin 14, is a type II classical cadherin predominantly expressed in the central nervous system, contributing significantly to axonal development and maintenance^38–40^. This study succeeded in documenting murine CDH18 expression in fetal heart explant culture, known to be derived from the epicardium. Given the thin-shaped nature of the epicardium, its density represents a notable minority in the context of the human heart. We did not detect protein expression in whole trypsinised whole-heart lysates, as mostly cardiomyocytes were isolated. In addition, our study suggests that *CDH18* expression shapes the fetal active epicardium transcriptome, but is downregulated in quiescent adult tissue^4,65^, thus explaining why *CDH18* remained undetected in the heart^40^.

Investigating the expression of CDH18 in human embryonic epicardial cells in vivo is technically difficult due to the lack of technical approaches to trace epicardial development as well as the limited availability of human fetal cells. The use of hiPSC overcomes this issue by enabling recapitulation of epicardiogenesis in vitro. Whereas our study limits our main conclusions to in vitro differentiated epicardial cells and their derivatives, we believe that our key discoveries are physiologically relevant, since RNA-Seq based meta-analysis shows that *CDH18* is expressed in the human heart during the third month of gestation, a time point of active development of the cardiovascular system in the human embryo, being in line with the hiPSC-based model shown in this study. Moreover, we documented murine embryonic CDH18 expression in epicardial explant cultures from E14.

Recent reports demonstrate an essential role for *WT1* in epicardial cell specification and maturation from the PE, suggesting that junction remodeling is crucial for this transition^66,67^. We showed that *CDH18* is expressed in correlation to *WT1* during epicardial development, leading us to hypothesize that *CDH18* might play a critical role in the specification and maintenance of epicardial cell identity, similar to *WT1*.

During human heart formation as well as during cardiac repair epicardial cells also contribute to coronary vessel formation by providing necessary vascular SMC and pericytes, which invade the myocardium. Notably, we provide compelling evidence to indicate that *CDH18* is critical for the modulation and establishment of epicardial-derived SMCs. We proved that *CDH18* downregulation modulates the expression of EMT markers and impacts epicardial identity. Recent studies characterized a *CDH18* tumor-suppressor role in glioma carcinogenesis and progression, demonstrating an active role in invasion and cell migration^38^, important processes that also relate to cardiac regeneration. The ectopic overexpression of *CDH18* was reported in relation to decreased migration capacity in accordance with our study. Most importantly, our study gained mechanistic insights into the biological regulation during the initiation of EMT in epicardial cells. The loss of *CDH18* decreased proliferation, *CDH1* expression and β-catenin. Interaction with and regulation of stabilization of β-catenin via cadherins is critical for the cellular organization, polarity and development^56,57^. Indeed, a previous study showed that Cdh14 interacts with and stabilizes β-catenin, similar to Cdh1^58^. Another study reported, that type II Cdh14 (Cdh18) along with type I E-cadherin (Cdh1) and N-cadherin (Cdh2) can bind to G12 family proteins^68^, which are known to regulate β-catenin translocation from the cell surface, a function associated with Rho-dependent cytoskeletal rearrangement. In our study, we demonstrated that the loss of CDH18 increased β-catenin levels, indicating Wnt activation and the promotion of EMT. The activation of Wnt signaling via β-catenin has long been deemed important for epicardial EMT and SMC differentiation which known to be regulated by Wt1^6,7,29,53,69,70^. Although controversial^52^, our study supports the necessity of β-catenin for SMC differentiation in vitro^70^.

*TCF21* is another key regulator of epicardial plasticity and EMT, promoting CF over SMC fate^60^. It is possible that rather than the activation of specific signaling pathways, the expression of TCF21 is of far greater importance for cell-fate decisions. Thus, investigating Wnt and TGF-β signaling pathways and their influence on *TCF21* could shed more light on this area, which will also allow understanding cell-fate decisions during cardiac regeneration, in particular those physiological stimuli that presumably force cells to assume a CF rather than pericyte phenotype. In addition, *TCF21* is known to be induced by retinoic acid (RA), thereby delaying SMC differentiation^60,71,72^. We showed that the expression of *ALDH1A2* is not upregulated until d24 and is low in d12 EPI cells. Thus, our observation of d24-silenced cells expressing lower levels of the SMC marker *ACTA2* could be linked to the higher presence of RA and *TCF21* in fetal-like cells compared to PE-like cells. During cardiac repair, the expression of *ALDH1A2* is re-activated, which might explain the preferred CF fate of epicardial cells that undergo EMT upon re-activation. Here, we showed loss of TCF21 upon loss of CDH18, suggesting a role for β-catenin-mediated Wnt activation in SMC differentiation. Furthermore, this observation occurred under the inhibition of the TGF-β co-receptor ALK5, which is known to impair EMT^73^. Our findings provide new insights into the regulation of the EMT process as a crucial step mediating epicardial-driven regenerative responses.

The exact mechanisms governing epicardial EMT and subsequent cell-fate decision of EPDCs in active epicardium are not well understood. Most experiments have used chick, avian or mouse models, which can differ considerably from humans. We observed the activation of downstream TGF-β signal targets, confirmed by RNA-Seq, upon *CDH18* silencing albeit treatment with SB431542. Our study endorses the link between TGF-β signaling and SMC phenotype^29,31,34,70^, as we observed SMC differentiation and TGF-β activation upon lower *CDH18* expression. Although *CDH18* overexpression could not completely block TGF-β-driven SMC differentiation, we found a partial prevention in the acquisition of SMC identity, indicated by lower levels of *ACTA2*. Altogether, our findings indicate a causal role of *CDH18* in TGF-β pathway during the acquisition of SMC identity.

CDH18 is a cell surface protein linked to G12 family proteins^68^, and RhoA pathway-mediated non-canonical TGF-β pathway, which has been linked to EMT and cell invasion^45,48–50,74,75^, might be activated upon the silencing. Interestingly, one study showed that inhibition of the RhoA pathway does not influence canonical TGF-β activation but still impairs SMC formation^76^. In our study, however, for those experiments that kept ALK5 inhibition conditions, ROCK inhibitor did not alter SMC marker expression upon *CDH18* silencing. Only when ALK5 inhibitor was absent, did inhibition of ROCK lead to a decrease in *ACTA2*.

Recently, a new study linked a TGFβ-independent causal role of the extracellular matrix (ECM) protein agrin to epicardial EMT mediated by dystroglycan^17^. This report also showed that their findings occur via Wnt signaling activation, a phenotype that could have common denominators with our study. Future studies are needed to shed more light into the different behavior of cells during human epicardial development. Such studies will also help deepen our understanding of epicardium re-activation upon cardiac injury and advance the field of cardiac regenerative medicine.

Finally, we demonstrated the correlative relationship between *CDH18* and *GATA4* expression, proposing *GATA4* as a putative regulator of *CDH18*. This effect likely occurs by static binding to the *CDH18* promoter near the transcriptional starting site, reinforcing *GATA4* as an important activator of the embryonic epicardium program.

CDH18 is a highly conserved protein in higher mammals and species that evolutionary developed a double closed circulatory system, maintaining an amino acid alignment of over 95% identity and 100% query sequence coverage relative to humans. The conserved sequence suggests that CDH18 has remained relatively unchanged far back up the phylogenetic tree in species developing a higher compartmentalized system to create a physical separation of oxygenated and deoxygenated blood (Supplementary Fig. 12). Higher demand for SMC might explain CDH18 conservation in development (Supplementary Fig. 13). Altogether, these findings indicate a specific role for CDH18 in epicardial regeneration and development, both processes that are under the control of evolutionarily conserved pathways

In conclusion, our work defines a biological function of *CDH18* in the epicardial context, enabling roads for the manipulation and therapeutic gene modulation of the active epicardium for cardiac repair and regeneration.

## Methods

### Cell lines and culture conditions

The hiPSC lines 201B7 and 409B2 (retroviral reprogramming by Yamanaka factors)^27^ as well as a MYH6-eGFP reporter cell line (MYH6-EIP4)^41^ were cultured in ReproCell ES media containing 4 ng/ml bFGF on irradiated MEF feeder cells. For removal of the feeder layer, the cells were treated with CTK. Feeder-free 201B7 were cultured in complete StemFit® AK02N media on iMatrix-511 (Matrixome) coated dishes. All cell lines used were female. MEC1 Mouse Embryonic Epicardial Cell Line SCC187 (Merck Millipore) was cultured and maintained in DMEM supplemented with 10% FBS. Mycoplasma testing was performed on a regular basis to exclude contamination.

### Epicardial induction

For epicardial differentiation, single cell suspension of hiPSC was generated using Accutase and subsequently cells were plated onto low-attachment HEMA-coated plates (6000-8000/96-well) to form embryoid bodies (EBs). Differentiation media was composed of complete StemPro®-34 media supplemented with 50 μg/ml ascorbic acid, 2 mM L-glutamine, 0.4 μM monothioglycerol and 150 mg/ml transferrin. To initiate differentiation, 0.5% Matrigel, 10 μM Y-27632 and 2 ng/ml human recombinant (hr)BMP4 were added to the differentiation media. After 24 h (h) EBs were cultured in differentiation media with a final concentration of 10 ng/ml hrBMP4, 2 ng/ml Activin A and 5 ng/ml hrbFGF. After 84 h, EBs were collected, dissociated using Accutase, and plated onto 0.1% gelatin-coated dishes (0.3 × 105 cells/cm^2^) in differentiation media containing 3 mM CHIR99021, 30 ng/ml hrBMP4, 5 ng/ml hrVEGF and 10 µM SB431542. From d7 onwards, induced hiPSC-derived epicardial-like (EPI) cells were maintained in maintenance media (DMEM containing 10% FBS and 10 µM SB431542). For cell passaging, EPI cells were detached by Accutase treatment for 5 min and replated as described above.

### Cell transfection

For the silencing experiments, Silencer^®^ Select siRNAs (ThermoFisher) were diluted to a 10 µmol stock solution: siCdh18#1 (s2816), siCdh18#2 (s2817), siGata4#1 (s535120) and siGata4#2 (s535121). To silence cells, 10 µl siRNA stock was diluted in 500 µl Opti-MEM, and 10 µl RNAiMax was added (silencing solution) followed by 10 min incubation at room temperature. The solution was added dropwise into one well of a six-well plate containing 2 ml maintenance media and 0.2–0.3 × 10^5^ cells seeded a day prior. The media was exchanged 24 h later, and the cells were cultured as usual thereafter. For the immunocytochemistry experiments, 260 µl silencing solution was added dropwise into one well of a 12-well plate containing 1 ml maintenance media and 0.05 × 10^5^ cells seeded a day prior to silencing.

For *CDH18* cDNA overexpression experiments, the cells were seeded to be 60–70% confluent at least one day prior to the transfection. For optimization of transfection protocol, transfection reagents and ratios as well as the plasmid amount were used as indicated in Supplementary Table 1. Unless otherwise stated, 6 µg plasmid per one well of a six-well plate or 34 µg plasmid per 10 cm dish were transfected using FuGENE^®^6 at an agent-to-DNA ratio of 2:1. Amounts of reagents were calculated using FuGENE^®^HD Protocol Database web tool. Cell sorting was performed by FACS via assessment of mCherry expression (Texas-Red positive cells).

### Induction of EMT

For the induction of EMT, EPI cells were passaged one day prior, at a density of 0.1–0.2 × 10^5^ per well of a six-well plate. For differentiation towards CF, the cells were treated with 10 ng/ml bFGF in 10% FBS DMEM for at least 8 days. For differentiation towards SMC cells were treated with 5 ng/ml TGF-β for 4 days followed by 10 ng/ml bFGF for another 4 days in 10% FBS DMEM.

### Isolation of mouse fetal hearts and whole-heart lysate

Embryos at E14 were collected into pre-warmed PBS and any extraembryonic tissue was removed using tweezers. Embryos were decapitated, the chest cavity was opened by ripping the anterior side apart and fetal hearts were isolated mechanically into a separate dish with pre-warmed PBS. Whole heart protein lysate was prepared by firstly cutting hearts into small pieces and treatment with trypsin for 20 min at 37 °C. The cell suspension was re-suspended, treated for an additional 20 min at 37 °C and supernatant was collected after centrifugation at 300 × *g* for 5 min.

### Ex vivo explant assay

Collected hearts at E14 were cut into 4–8 pieces and placed onto 0.1% gelatin-coated dished in DMEM (low glucose) supplemented with 15% FBS. Outgrowth cultures formed as early as 24 h post placing. After 72 h heart pieces were manually removed with tweezers. Explant cells were passage by trypsinization the following day and cultured in 10% FBS-DMEM (low glucose) supplemented with 10 µM SB431542. Cells were collected 1–3 days after passage. EMT was induced as described above in 10% FBS-DMEM (low glucose).

### Quantitative RT-PCR

For RNA extraction, live cells were collected as du- or triplicate in QIAzol lysis reagent, and total RNA isolation was performed using the RNAeasy micro kit (Qiagen) according to the manufacturer’s manual. The generation of cDNA was performed by using the ReverTra Ace system (Toyobo BIOTECH) according to the manufacturer’s manual. QRT-PCR was performed in du- or triplicate either using TaqMan gene expression assays (*WT1* Hs01103750_g1; *TBX18* Hs01385457_m1; *ALDH1A2* Hs00108254_m1; *TCF21* Hs00162646_m1) in TaqMan master mix solution or SYBR green (ThermoFisher) with primers against identified candidates (Supplementary Table 2) in SYBR green master mix solution according to the manufacturers’ respective manuals. Data acquisition was carried out by StepOne Plus (AppliedBiosystems). Analysis was performed using Microsoft Excel and GraphPad Prism. Values without readout were not included. The distribution of values was verified by a box plot depiction to determine the appropriate statistical analysis. For direct comparison of dataset normally distributed data were analyzed by students t-test, as indicated in figure legends, otherwise, the Mann–Whitney test was used. Comparisons of more than two datasets were carried out via ANOVA, according to experimental conditions, as indicated in the figure legends. All error bars represent the standard error of the mean (SEM). For co-expression analysis, qRT-PCR data was used to generate a regression line by GraphPad Prism v7 and higher.

### Immunocytochemistry

Cells were fixed by 4% PFA treatment for 15 min and stored in PBS at 4 °C. Blocking was performed for 30–45 min in blocking buffer: 1% BSA and 0.5% Triton X for nuclear staining or 0.1% Tween 20 for surface or intracellular staining and 0.1 M glycine in PBS. Following three PBS washes, primary antibody was added in blocking buffer without glycine and incubated overnight at 4 °C. The primary antibodies used are as follows: Anti-WT1 1:200 (Abcam; ab89901), Anti-ZO1 1:100–300 (Invitrogen; ZO1-1A12), Anti-POSTN 1:200 (ThermoFisher; PA5-98301), Anti-α-SMA 1:200 (Abcam; ab7817) and Anti-Ki67 1:200 (BioLegend; 350502). The next day, the cells were washed thrice with PBS, secondary antibody (Invitrogen, 1:1000–2000) was added in 1% BSA-PBS and incubated for 2 h at room temperature. The secondary antibodies were used are as follows: goat-anti-mouse-Alexa488 (A11001), goat-anti-rabbit-Alexa546 (A11010), goat-anti-mouse-Alexa546 (A11030), goat-anti-mouse-Alexa594 (A11032), goat-anti-rabbit-Alexa647 (A21245), goat-anti-mouse-Alexa647 (A21236). Following three washes with PBS, Hoechst or DAPI (5 ng/ml, 1:10,000) was added for nuclear counterstaining.

### Flow cytometry analysis/FACS

The cell was dissociated into single-cell suspension by Accutase treatment, washed twice and fixed using 4% PFA for 15 min. For nuclear staining, the cells were permeabilized by 0.1% Triton X solution. Conjugated antibodies were diluted 1:50 in FACS buffer (5% FBS-PBS) and incubated at room temperature for 30–45 min. The conjugated antibodies used are as follows: WT1-Alexa488 (Abcam; ab202635), rb-IgG-Alexa-488 (Abcam; ab199091), CDH18-FITC (Biorbyt Ltd; orb7854), CD22-APC (BD; 562860) CD31-APC (BioLegend; 303116) and CD144-FITC (BD; 560874).

For sorting, a single cell suspension was generated as described above, and counterstaining with 1:1,000 DAPI (5 ng/ml) was performed for dead cell exclusion. Sorting gates were set as described.

Analysis was performed using BD FACSDiva v6 or v8 and FlowJo v10. The cell population was identified by FSC/SSC gating and doublets discrimination was performed. Negative gates were established using unstained samples or isotype controls and negative control samples. Positive gates were set to contain no negative signal.

### Immunoblotting

Cells were detached using Accutase and lysed in Mammalian Protein Extraction Reagent (M-PER) (Thermo Scientific; 78501) buffer. The amount of protein was determined by the Bradford assay using BSA as the standard. The primary antibodies (dilution 1:1000) used are as follows: Anti-CDH18 antibody (Proteintech 13091-1-AP), Anti-GATA4 (CST; 369665S (D3A3M)), Anti-SNAI1 (Abcam; ab63371), Anti-α-SMA (Abcam; ab11952), Anti-β-actin (Sigma; A5441), Anti-WT1 (Abcam; ab89901), Anti-CD22 (Abcam; ab207727), Anti-TCF21 (Abcam; ab32981), Anti-β-catenin (CST; 8814), Anti-phosporylated-β-catenin (S33/S37/T41) (CST; 9561), Anti-TNNI1 (Abcam; ab203515). The secondary antibodies (dilution 1:5000) used were as follows: goat anti-rabbit-horseradish peroxidase (HRP) (Abcam; ab97051), rabbit anti-mouse-HRP (Abcam; ab97046). Blots derive from the same experiment and were processed in parallel. Unprocessed blots are shown in Supplementary Figs. 14–18.

### Proliferation assay

A time-course curve of parental (scr) and siRNA-expressing cells was generated by seeding 4.5 × 10^3^ cells into 6-cm bottom-well diameter dishes. After 24 h, fresh medium was replaced and cells were fixed to be referred as a standard for relative growth (day 0). Relative growth was assessed every 48 h (2 days), fixing in 2% PFA and stained with 1% crystal violet (Sigma; C6158-50G). After extensive cell washing, crystal violet was solubilized in 20% acetic acid (Sigma) and quantified absorbance at 595 nm as a relative assessment of cell number (PerkinElmer; EnVision 2104 Multilabel Reader). Relative values in Fig. 4 represent cell growth of daughter the indicated medium. Percentage zero (0%) refers to initial growth at day 0.

### Invasion assay

Cell were silenced as described above and collected 4 days later. 2.5 × 10^4^ cells were seeded onto a Corning^®^ Biocoat^®^ Matrigel® Invasion chamber and incubated for 24 or 48 h. The removal of non-invasive cells was carried out as stated in the Corning^®^ Biocoat^®^ Matrigel^®^ Invasion manual, and the cells were fixed for 2 min in methanol followed by staining using crystal violet overnight and finally washed with distilled water. Pictures were taken using BZ-X710 (Keyence) and cell counting was performed with ImageJ. Cell counting was performed blinded using ImageJ v1.52. Images were converted to 8-bit greyscale format, and the same threshold was set for all images to highlight cell structures. Potential cell clusters were separated by the watershed function, and only structures 0.01-0.1 pixel^2^ in size were counted. Graphs were generated using GraphPad Prism v8.

### Bioinformatics

Retrospective analysis of *CDH18* gene expression was done using GSE84085 (GEO) for epicardium development and GSE7307 for cardiovascular-derived tissues (subset of aorta, coronary artery, heart, heart atrium and heart ventricle) [GEO and R2; genomic analysis and visualization platform (http://r2.amc.nl/)].

Promoter sequences were identified by *Ensembl* and then screened for transcription factor binding sites with PROMO-ALGGEN (based on TRANSFAC v8.3) and JASPAR 2016.

### Image acquisition, processing and analysis

Microscopy images were taken by BZ-X710 and processed by BZ-X Analyzer (Keyence); pseudo-coloring was used as indicated in figure legends. For phase-contrast and fluorescence pictures, a representative section was chosen, cropped and magnified. Western blot data were recorded by LAS4000 (Cytiva). General image analysis was performed using Microsoft powerpoint as well as ImageJ v1.52.

### RNA-sequencing

Data normalization was carried out using NOISeq. The final processed data and raw fastq files generated de novo in this study were submitted to Gene Expression Omnibus (GEO) under the accession code GSE165450. The raw data were analyzed by using RStudio for further analysis on gene expression. Gene expression data for reading, exploring and pre-processing were conducted using the Bioconductor package *NOISeq* pipeline to perform data and differential expression analysis for RNA-Seq. A hierarchical derived cluster dendrogram was generated by using hclust, *stats* package, and *agnes* in the *cluster* package. Distances were assessed by using Manhattan city-block distance algorithm. *K*-means were calculated with the kmeans function. Distance and correlation matrices were computed and plotted by using *get_dist*, *fviz_dist* included in the *factoextra* package. The *fviz_cluster* function was used to compute cluster scatter plots. Heatmaps were depicted to cluster the expression data for DEGs using R script. Statistical analysis and visualization of the functional profiles of genes as well as gene clusters for GO terms were conducted using the *DOSE* and *clusterProfiler*. Ingenuity pathway analysis (IPA) was used to develop an upstream pathway analysis as well as pathway activity patterns. Chord diagrams were generated by using GOplot.

### Material availability

The CDH18 overexpression plasmid used in this study is available from VectorBuilder [VB200615-1011cyh]. Other materials are available from the corresponding author upon reasonable request, but we may require payment and/or a Materials Transfer Agreement.

### Animal experiments

All experiments involving animals were approved by the Kyoto University Animal Experimentation Committee and carried out in accordance to the Guidelines for Animal Experiments of Kyoto University and Guide to the Care and Use of Laboratory Animals by the Institute of Animal Resources.

### Reporting summary

Further information on research design is available in the Nature Research Reporting Summary linked to this article.

## Supplementary information

Supplementary Figures and Tables

REPORTING SUMMARY

## Data Availability

All data supporting this study are available within this publication and its supplementary information or can be provided upon request. The RNA-Seq dataset generated in this study is deposited at Gene Expression Omnibus (GEO) with the accession code GSE165450.
