# Supplementary material for: CDH18 is a fetal epicardial biomarker regulating differentiation towards vascular smooth muscle cells
Source: NPJ Regen Med. 2022 Feb 2;7:14. doi: 10.1038/s41536-022-00207-w (PMC8810917; doi:10.1038/s41536-022-00207-w)
Supplement: Supplementary file 1 — Supplementary Figures and Tables [file 41536_2022_207_MOESM1_ESM.pdf]

Supplementary Figure 1

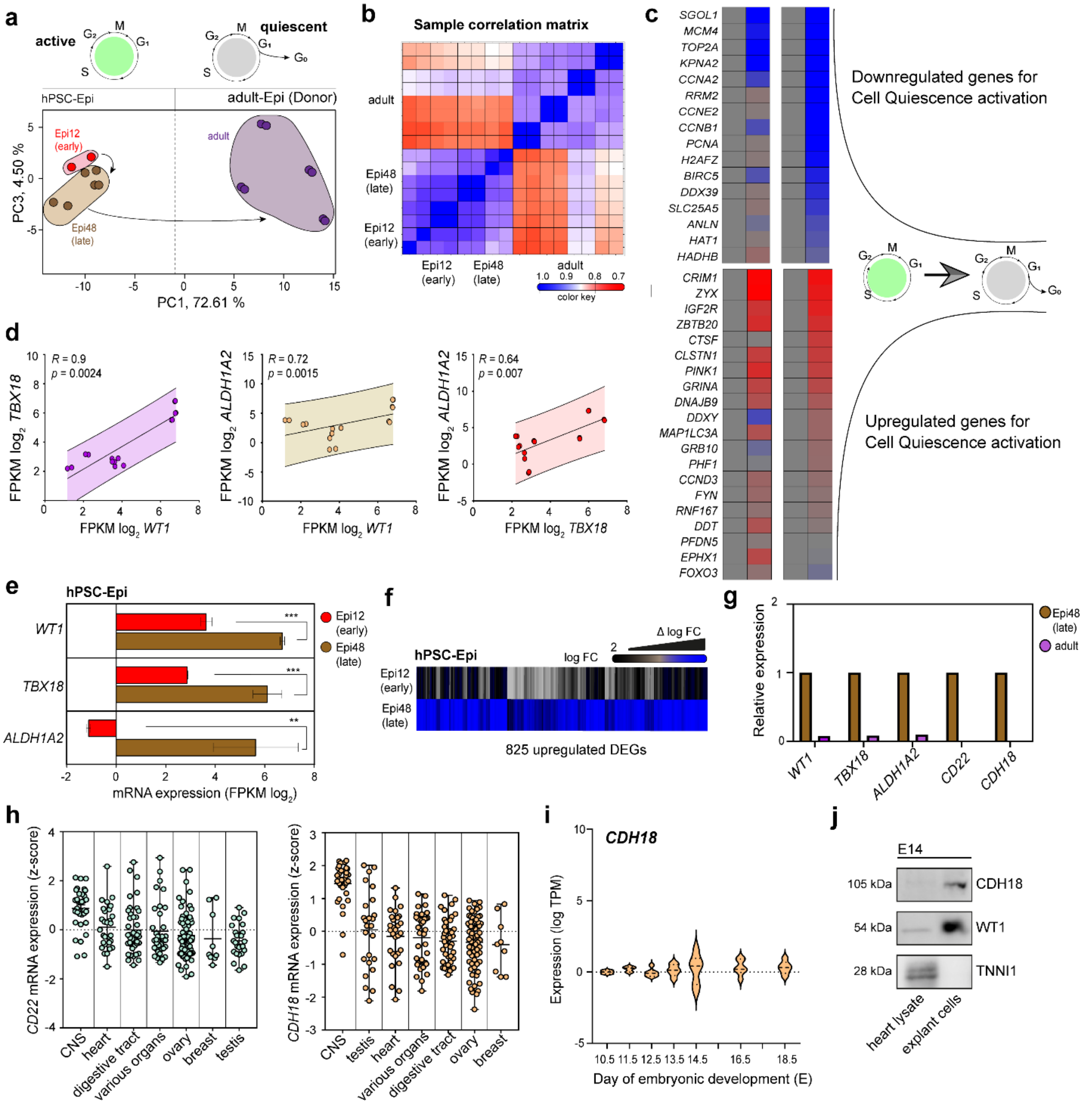

Supplementary Figure 1. Transcriptional profiling of active epicardial cells for cell surface markers

a PCA using the GSE84085 RNA-Seq expression dataset of d12 early epicardial-like cells (Epi12, red) [19-9-7-Epi, n=2], d48 late epicardial-like cells (Epi48, brown) [H9-Epi, ES03-

Epi and 19-9-11-Epi, *n*=6] and human adult epicardium (dark violet) [donors 9605, 9633, 9634 and 9635, *n*=8]. **b,** Sample correlation matrix resulting from the consensus clustering analysis of mRNA data, shows transcriptomic differences among samples. **c,** Retrospective analysis showing a heatmap of quiescence marker genes in adult epicardium (adult) compared to embryonic induced epicardial cells Epi12 (right) and Epi48 (left). **d,** Transcriptomic correlative analysis (Pearson, R) of genes defining the embryonic epicardium. **e,** Analysis of *WT1*, *TBX18* and *ALDH1A2* expression Epi12 (red) and Epi48 (brown) [* $p<0.05$; ** $p<0.01$; *** $p<0.001$, students' t test]. **f,** DEG analysis to identify genes that are at least 2-fold upregulated in the active epicardium. **g,** Fold change of active epicardium markers and identified candidates in adult tissue (dark purple) relative to Epi48 (brown). **h,** Ranked *CD22* (left) and *CDH18* (right) expression by tissue (z-score) [GSE7307]. **i**, GSE1479 retrospective analysis for *CDH18* mRNA expression in mouse heart embryonic development [*n*=36; E10.5 = 3, E11.5 = 3, E12.5 = 6, E13.5 = 6, E14.5 = 6, E16.5 = 6, E18.5 = 6] [One-way ANOVA]. **j**, Western blot analysis of CDH18, WT1 and cardiac troponin 1 (TNNI1) expression in murine fetal whole-heart lysate and fetal heart explant culture.

[data shows GSE84085 unless stated otherwise]

Supplementary Figure 2

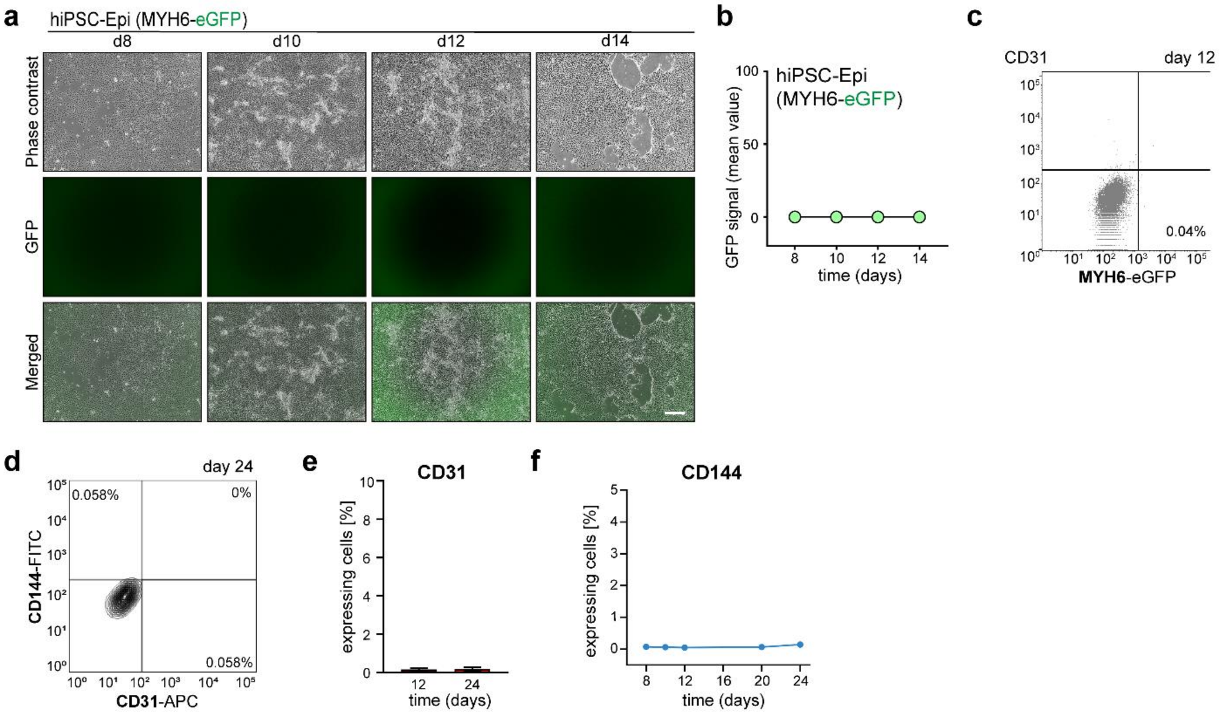

Supplementary Figure 2. Exclusion of cardiomyocyte traces

a, Phase contrast and fluorescent microscopy of EPI cells induced from MYH6-eGFP reporter line at d8-14 showing no visible eGFP expression [scale bar 200µm]. **b, c**, Flow cytometry analysis of **b** eGFP expression over time and **c** CD31 and eGFP expression in d12 EPI cells [*n*=3]. **d-f**, Detection of CD31 and CD144: **d** contour blot of d24 EPI cells; **e** CD31 expression in d12 and d24 EPI cells and **f** CD144 expression over time of induction.

Supplementary Figure 3

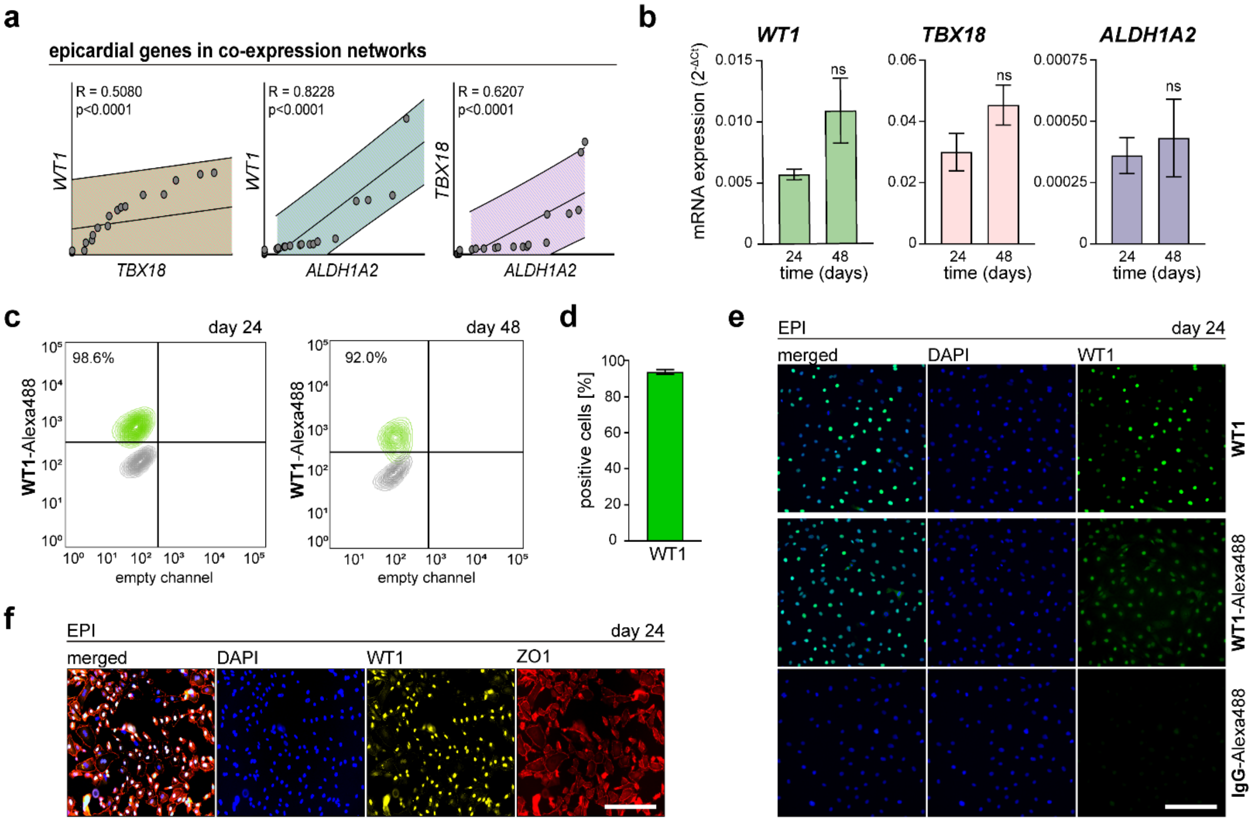

Supplementary Figure 3. Molecular characterization of EPI cells

a, Transcriptomic correlative analysis (Pearson, R) of genes defining the fetal stage of epicardial development in EPI cells based on (Fig. 2c). **b**, qRT-PCR analysis of epicardial markers *WT1* (light green), *TBX18* (light red) [d24 *n*=8, d48 *n*=10] and *ALDH1A2* (light purple) [d24, d48 *n*=8]. There was no statistically significant difference (ns) between d24 and d48 [Mann-Whitney-test; error bars indicate standard error of the mean (SEM)]. **c**, Flow cytometry-derived contour blot of d24 (left) and d48 (right) EPI cells showing WT1 expression (green) [gray, unstained control]. **d-f**, EPI d24 cells showing WT1 expression and **d** its quantification of positive cells counted in ImageJ. **e** Immunofluorescence staining of cells was performed using both purified and conjugated WT1 antibody to

confirm positivity of cells. **f** EPI cells display cobblestone-like cell morphology and the expression of ZO1 and WT1. DAPI staining marks cell nuclei [scale bar 100µm; images were cropped to show a representative section].

Supplementary Figure 4

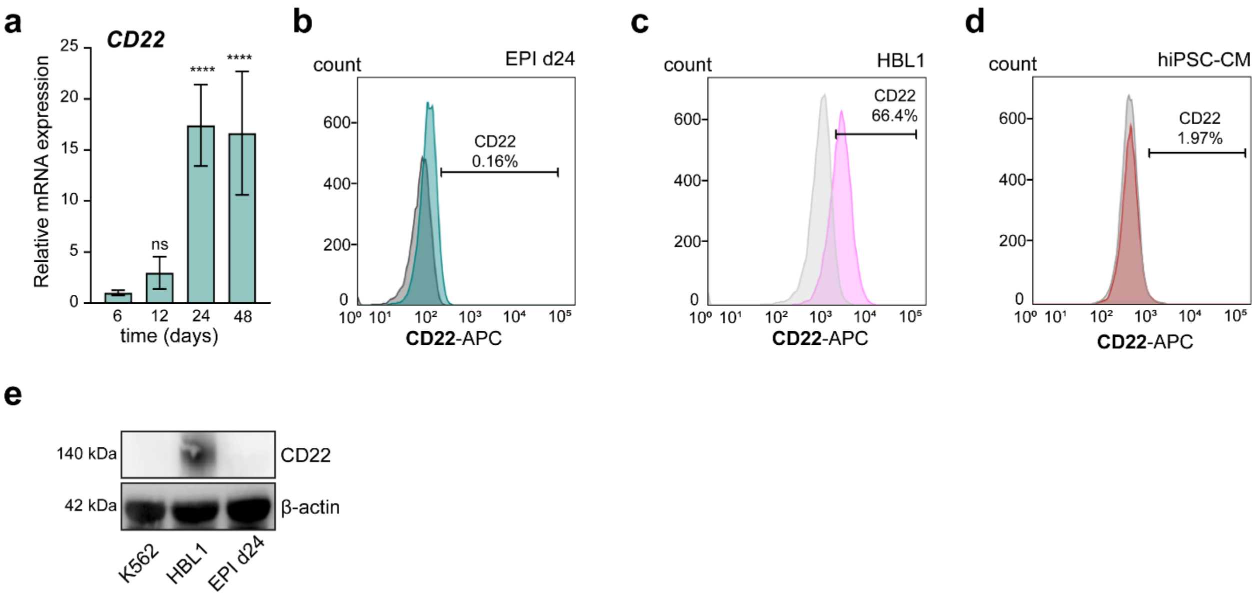

Supplementary Figure 4. Evaluation of CD22 expression

a, qRT-PCR analysis of *CD22* (deep turquoise) during induction normalized to d6. [d6 *n*=4, d12 *n*=4, d24 *n*=8, d48 *n*=8; **** $p<0.0001$]. **b**, Flow cytometry analysis for CD22 (deep turquoise) [gray, unstained control]. **C,d**, Flow cytometry analysis for CD22 in **c** HBL1 cells (light pink) acting as a positive control and **d** hiPSC-derived CM (hiPSC-CM) (dark red) acting as a negative control [grey, unstained control]. **e**, Western blot analysis for CD22 in EPI cells at d24 with HBL1 cells acting as a positive control and K562 cells acting as a negative control.

Supplementary Figure 5

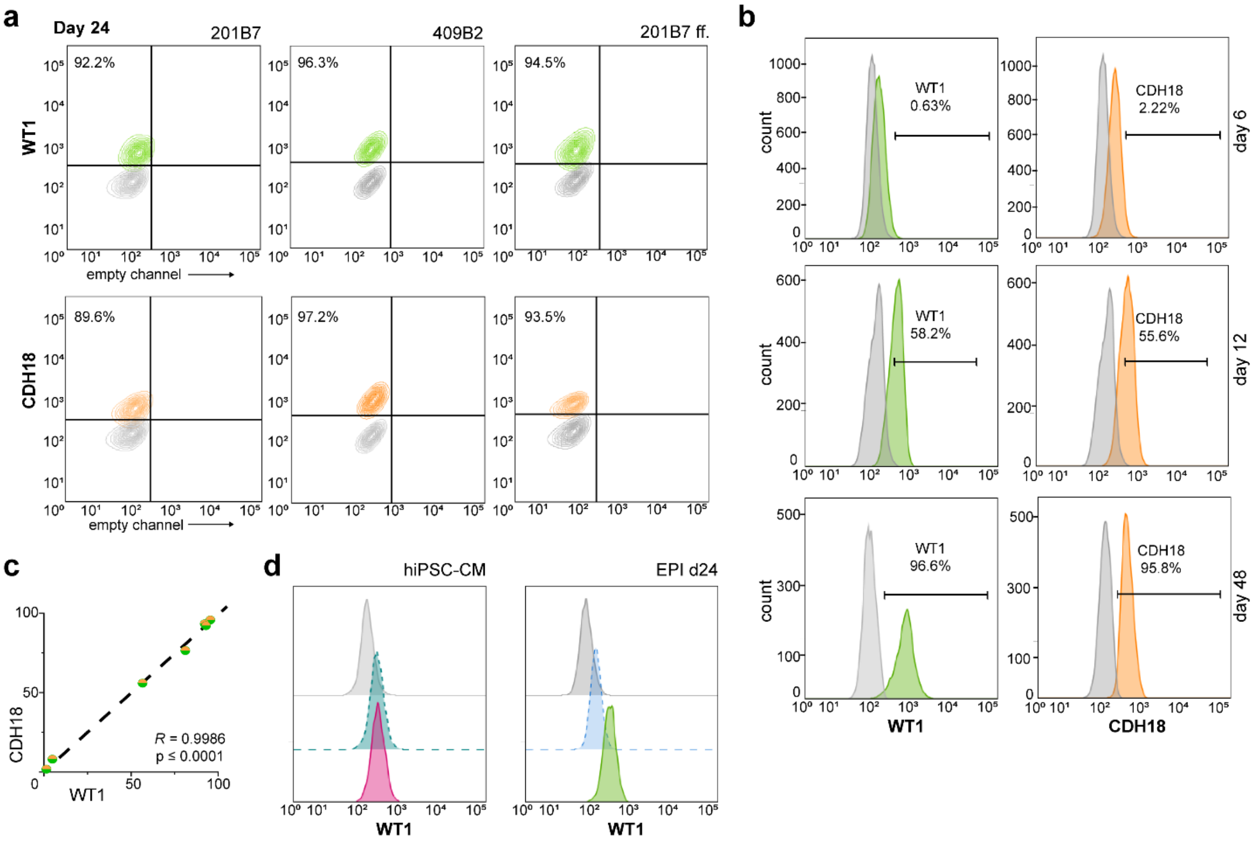

Supplementary Figure 5. Evaluation CDH18 as a putative biomarker in epicardiogenesis

a, Contour blots of d24 EPI cells showing WT1 (upper panel, green) and CDH18 (lower panel, orange) expression in cells differentiated from 201B7 and 409B2 maintained on-feeder and 201B7 maintained feeder-free (ff.). **b**, Histograms depicting WT1 (left, green) and CDH18 (right, orange) expressions detected by flow cytometry in d6, d12 and d48 EPI cells. **c**, Correlation analysis (data Fig. 2H) for WT1 and CDH18 expression. **d**, Half-Offset overlay histograms of WT1 expression in hiPSC-CM (dark pink) showing no WT1 expression and EPI cells at d24 (green) [dotted lines mark isotype control, turquoise: hiPSC-CM; light blue: EPI; gray: unstained cells].

Supplementary Figure 6

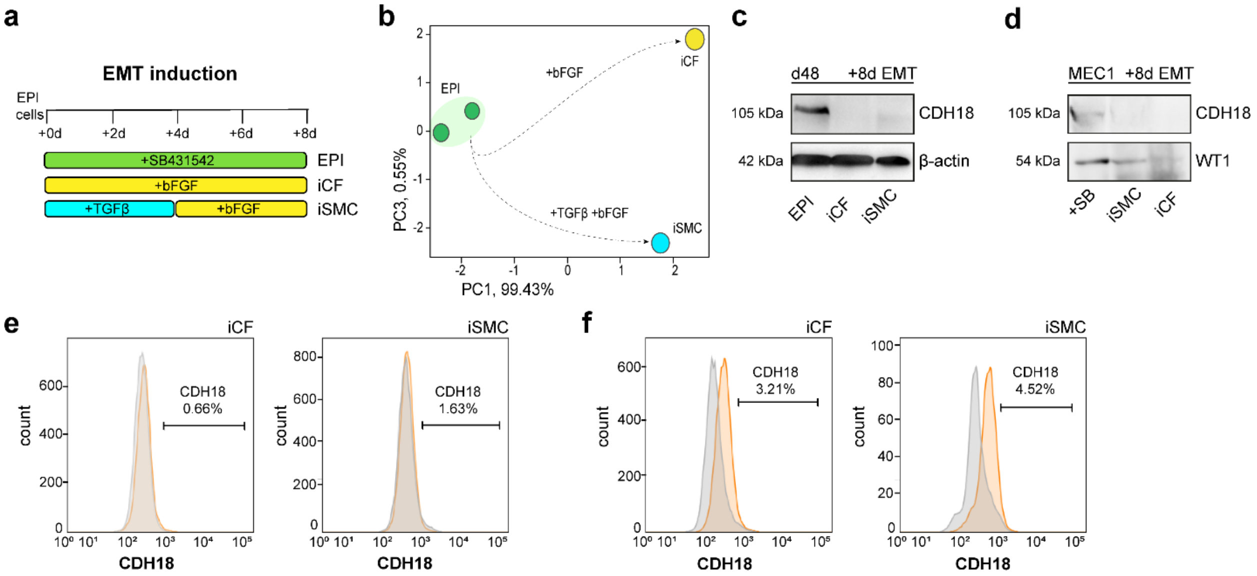

Supplementary Figure 6. CDH18 expression in EPDCs

a, Scheme of EMT induction for the directed differentiation of induced CF (iCF) and induced SMC (iSMC). **b**, PCA plot of EPI cells at d24, induced CF (iCF) and SMC (iSMC) [GSE165450]. **c**, Western blot analysis for CDH18 expression in EPI cells and derivatives derived from d48. **d**, CDH18 and WT1 expression analysis in MEC1 cell line treated accordingly to EMT induction scheme described in **a** [+SB = +SB431542]. **e, f**, Histogram showing CDH18 expression in EPDCs derived from **e** d12 and **f** d24 EPI cells.

Supplementary Figure 7

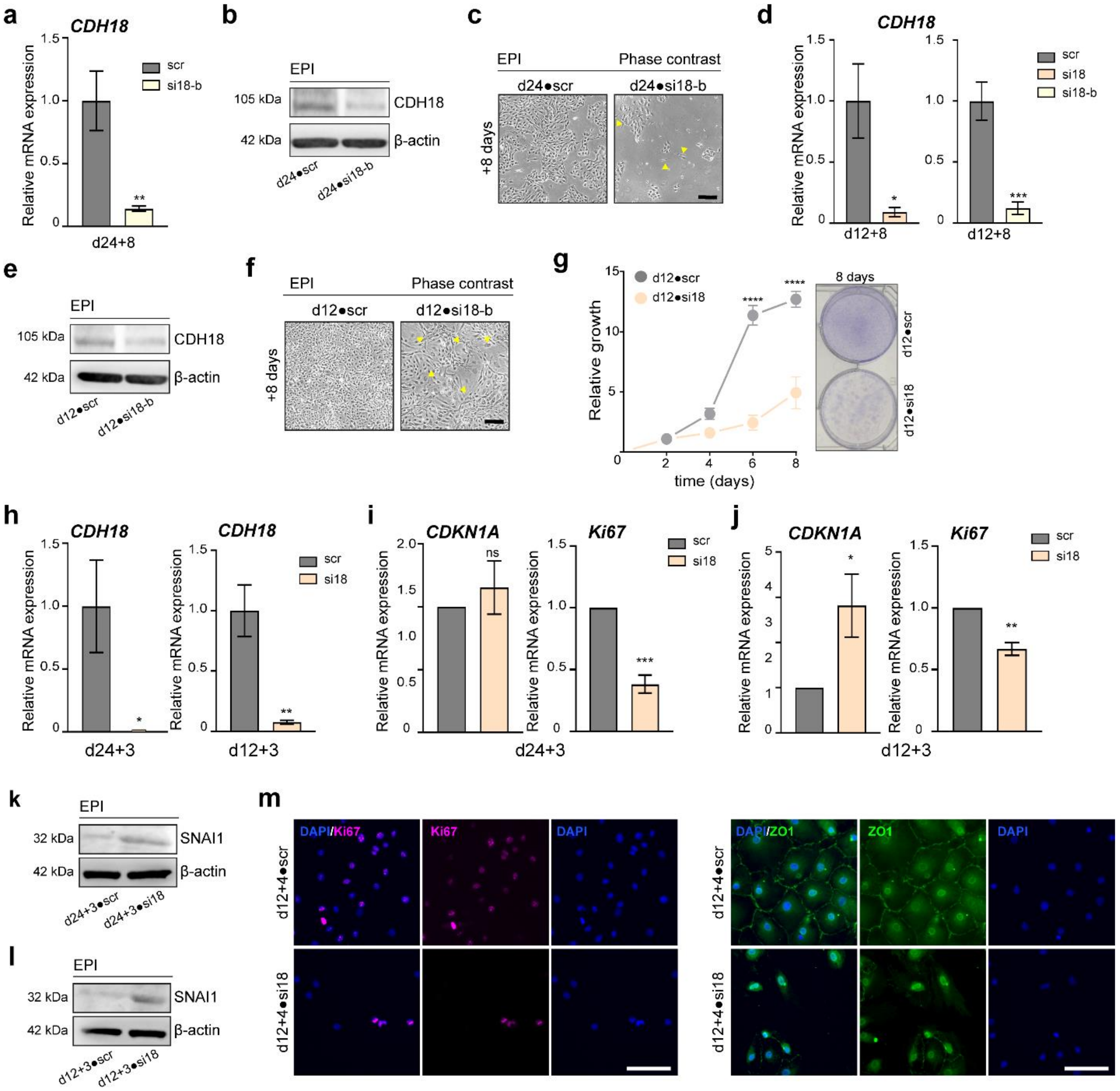

Supplementary Figure 7. Downregulation of *CDH18* leads to loss of epicardial identity and initiation of EMT

a-c, *CDH18* downregulation by a second siRNA against *CDH18* (si18-b) in d24 EPI cells: **a** Reduction of mRNA expression [*n*=3; ** *p*=0.0048] and **b** protein expression and **c** changed morphology, i.e., elongated cells (yellow arrowheads). **d**, Knockdown

verification 8 days after silencing using two different siRNAs against *CDH18* in d12 EPI cells [*n*=3; * *p*=0.014, *** *p*=0.0003]. **e, f**, Validation of **e** reduced CDH18 and **f** changes morphology (yellow arrowheads) in si18-b-treated cells after 8 days. **g**, Growth curve of d12 cells silenced for *CDH18* and control (scr) for 2-8 days [*n*=3; **** *p*<0.0001]. **h**, Knockdown verification 3 days after silencing of *CDH18* in d24 (left) and d12 (right) EPI cells [*n*=3; * *p*=0.0222, ** *p*=0.0016]. **i, j**, Downregulation of proliferation markers 3 days after silencing in **i** d24 and **j** d12 EPI cells [scr normalized to 1 for each experiment; *n*=3; paired Students t-test: * *p*=0.0483, ** *p*=0.0013, *** *p*=0.0004, ns= not significant]. **k, l**, Western blot analysis for SNAI1 detection upon 3 days after silencing in **k** d24 and **l** d12 EPI cells. **m**, Immunofluorescence staining of d12 EPI cells 4 days after silencing for Ki67 and ZO1 expression.

[scale bars = 100µm; statistical analysis performed by Students t-test unless indicated otherwise, error bars represent SEM].

Supplementary Figure 8

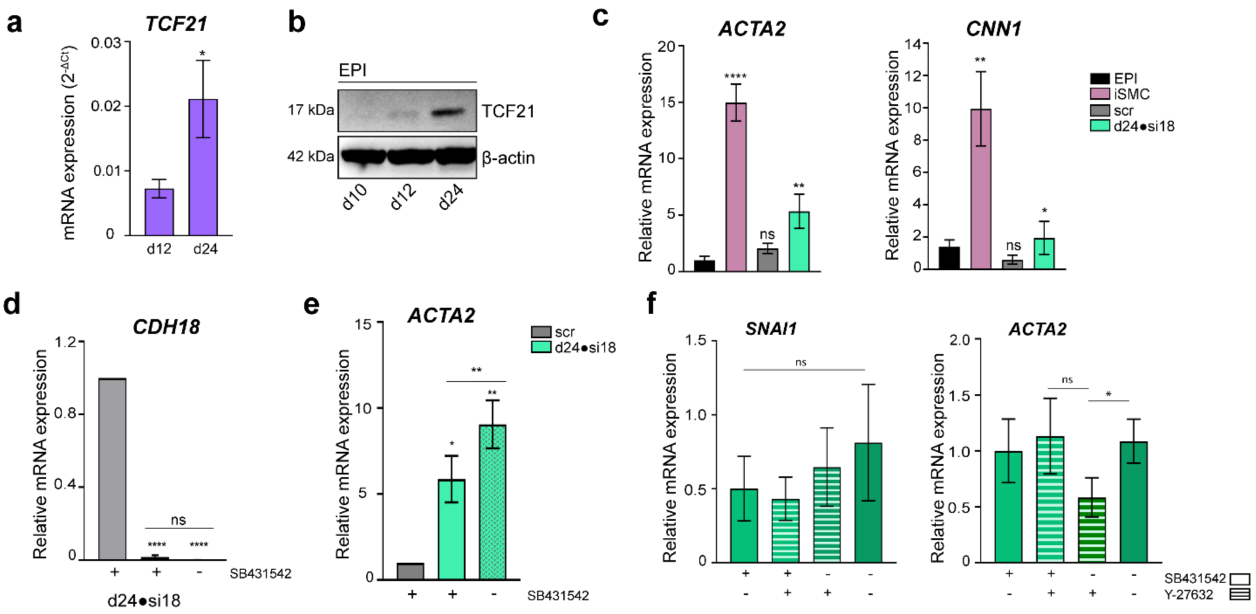

Supplementary Figure 8. Downregulation of *CDH18* induced differentiation into SMC

a, b, Expression levels for *TCF21* from d12 to d24 based on **a** qRT-PCR [n=4] and **b** western blot analysis. **c**, Expression of SMC markers in d24 EPI cells either silenced (si18) or induced towards SMC (iSMC) for 8 days. Cells displayed an upregulation of *ACTA2* (left) and *CNN1* (right) in both iSMCs and si18 cells but not in EPI or scr-treated cells, albeit upregulation in d24•si18 was only modest. Data is normalized to EPI cells [n=3; Students t-test: * p<0.05, ** p<0.009, *** p<0.0005, **** p<0.0001]. **d, e**, Expression of **d** *CDH18* and **e** *ACTA2* in d24 silenced cells in presence or absence of TGFβ-inhibitor (SB431542) [scr normalized to 1 for each experiment; n=3; RM-one-way ANOVA; * p<0.05, ** p<0.009, **** p<0.000]. **f**, Expression of *SNAI1* (left) and *ACTA2* (right) in d12 silenced cells in presence or absence of TGFβ-inhibitor (SB431542) and ROCK inhibitor

(Y-27632) [n=3; RM-one-way ANOVA with Tukey’s multiple comparison test; * $p<0.0367$].

[error bars indicate SEM].

Supplementary Figure 9

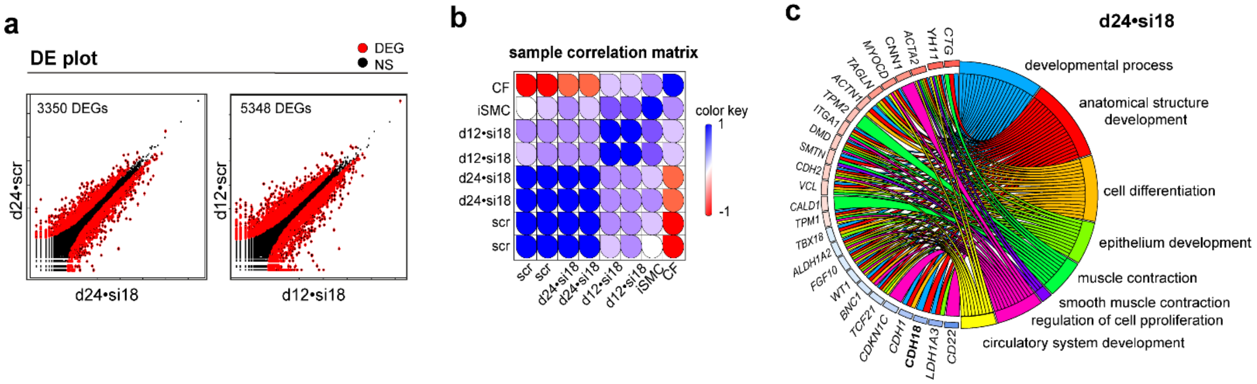

Supplementary Figure 9. Transcriptional profiling of *CDH18*-silenced EPI cells

a, Differential expression (DE) plot showing the number of DEGs (red dots) in *CDH18*-silenced d24 EPI (d24•si18, left) and d12 EPI (d12•si18, right) cells. Black dots indicate genes without significantly different expression (NS). **b**, Sample correlation matrix resulting from the consensus clustering analysis of mRNA data and showing transcriptomic differences among samples [GSE165450]. **c**, Chord diagram representing flow and detailed relationship between gene expression levels of d24•si18 DEGs (left semicircle perimeter) and their enriched GO biological processes (right semicircle perimeter) [GSE165450].

Supplementary Figure 10

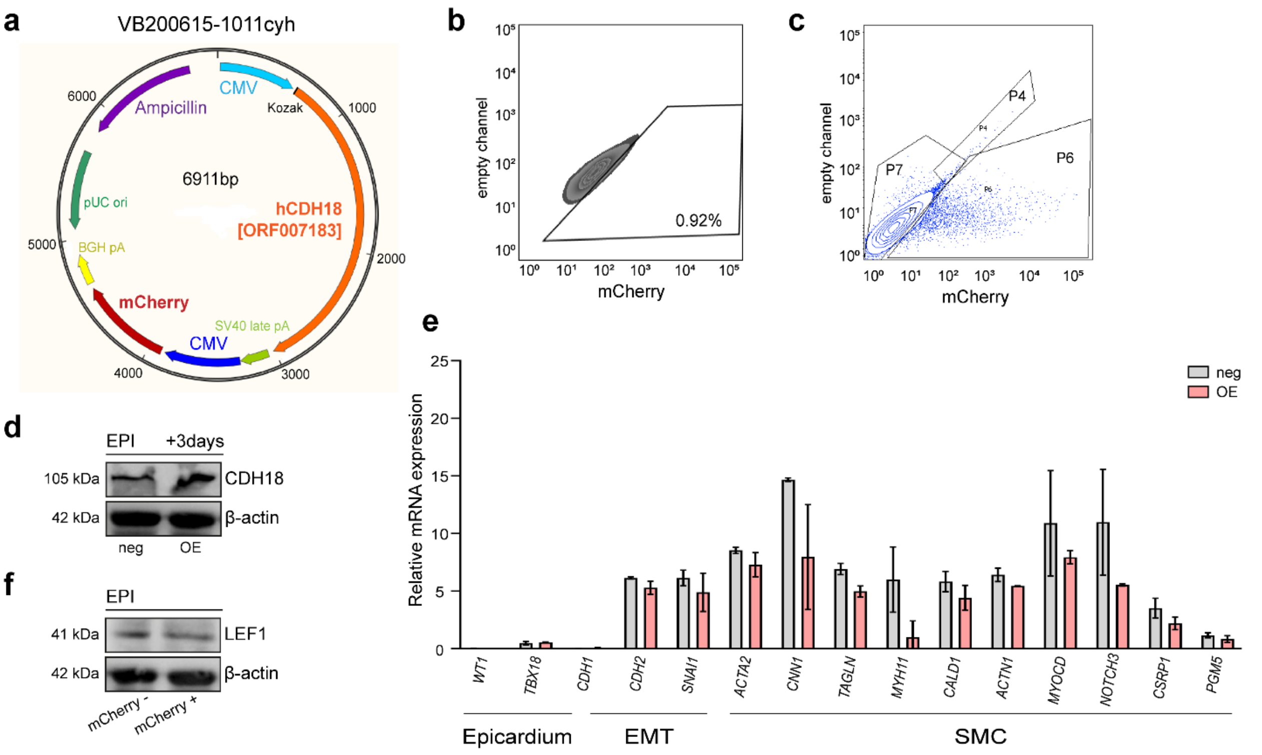

Supplementary Figure 10. ***CDH18*** **overexpression in EPI cells**

a, Vector map generated using SnapGene® of *CDH18* overexpression plasmid designed using the VectorBuilder platform [VB200615-1011cyh], containing human *CDH18* cDNA under a CMV promotor as well as mCherry-expression cassette for transfection validation. **b**, Flow cytometry analysis of non-mCherry expressing (mCherry-) cells. **c**, FACS gating: The P6 population was classified as mCherry+, P7 as mCherry-. The P4 population was defined as highly auto-fluorescent cells and therefore excluded from sorting. **d**, Knockdown verification 3 days after transfection in transfected (OE) and empty transfected (neg) cells. **e**, Expression analysis of RNA-seq dataset [GSE165450] of characteristic signature genes in OE and neg cells. **f**, LEF1 expression in mCherry + sorted and mCherry – sorted cells.

Supplementary Figure 11

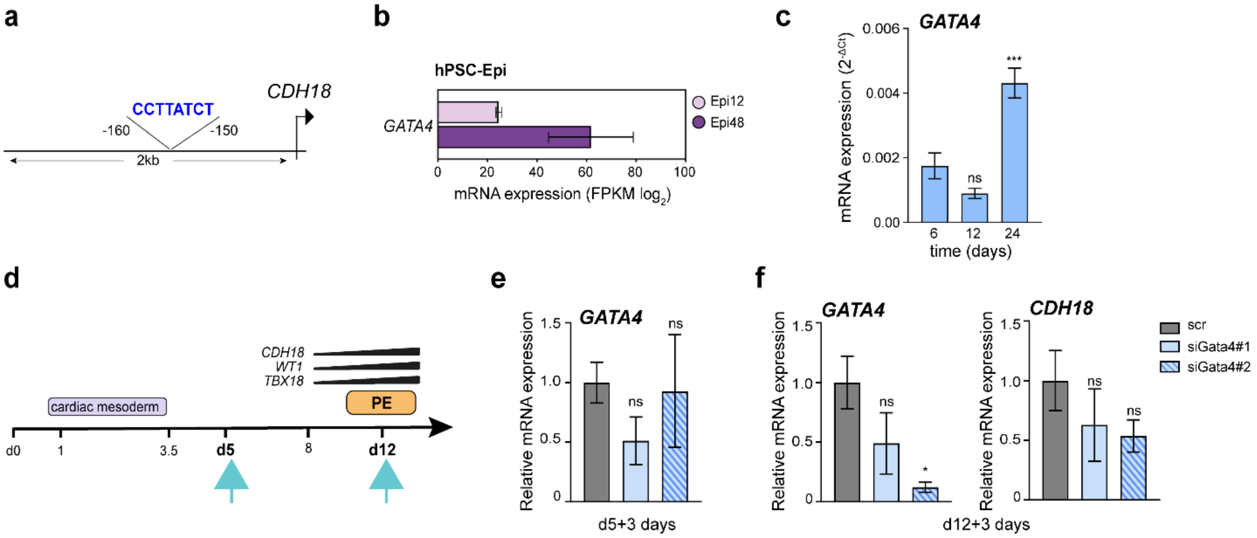

Supplementary Figure 11. Characterization of *GATA4* expression during epicardial formation

a, *CDH18* promotor region (2 kb) with the predicted binding sequence of *GATA4* identified by binding site analysis. **b**, *GATA4* expression in Epi12 and Epi48 cells [GSE84085]. **c**, Time course of *GATA4* expression during EPI induction analyzed by qRT-PCR [d6-12, *n*=4; d24 *n*=8; *** *p*=0.007]. **d**, Scheme of the silencing experiments during EPI induction. **e, f**, Day 3 after silencing using two different siRNAs (siGata4#1 and siGata4#2) in **e** d5 EPI cells showing *GATA4* expression and **f** d12 EPI cells showing *GATA4* and *CDH18* expression [*n*=3; ns (non-significant), * *p*<0.05].

[statistical analysis performed by one-way ANOVA with Dunnett's multiple comparison test; error bars represent SEM].

Supplementary Figure 12

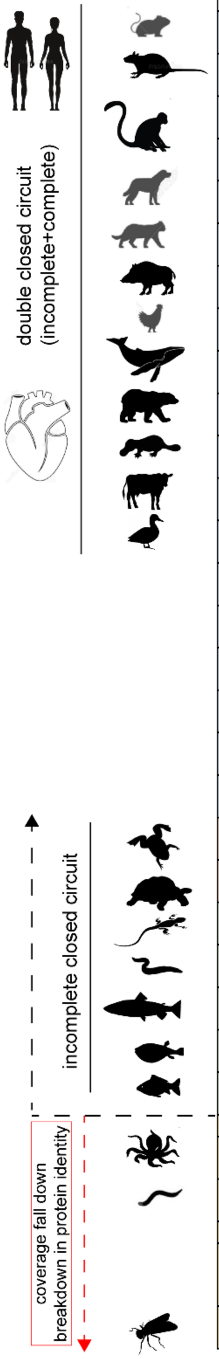

Organism	Protein name	Query coverage [%]	Identity [%]	Homo-/Orthologous	NCBI	Uniprot	Circulatory system
Mus musculus	cadherin-18 precursor	100	96.08	Yes/Yes	NP_001074768.1	E9Q9Q6	double closed
Rattus novergicus	cadherin-18 precursor	100	96.71	Yes/Yes	NP_001101126.2	F1M702	double closed
Pan troglodytes	cadherin-18 isoform X1	100	99.62	Yes/Yes	XP_024212431.1	H2QQP5	double closed
Pongo abelii	cadherin-18 isoform X1	100	99.37	Yes/Yes	XP_024103414.1	H2PF84*	double closed
Canis lupus familiaris	cadherin-18 isoform X1	100	97.22	Yes/Yes	XP_013968634.1	F6Y5X3	double closed
Felis catus	cadherin-18 isoform X4	100	97.22	Yes/Yes	XP_019668046.1	M3WJ78	double closed
Sus scrofa	Cadherin-18 isoform X1	100	97.59	Yes/Yes	XP_020932622.1	A0A287AN03	double closed
Gallus gallus	cadherin-18 isoform X4	100	91.77	Yes/Yes	XP_426046.4	A0A1D5PZT9	double closed
Balaenoptera acutorostrata scammoni	cadherin-18 isoform X1	100	96.58	Yes/Yes	XP_007188294.1	A0A384ALT3_BALAS	double closed
Ursus maritimus	cadherin-18 (predicted)	100	96.96	Yes/Yes	XP_008688109.1	A0A384C0Z5	double closed
Ornithorhynchus anatinus	cadherin-18	100	89.57	Yes/Yes	XP_001507828.2	F6VFP8	double closed
Bos taurus	cadherin-18 precursor	100	97.08	Yes/Yes	NP_001070305.1	Q08DJ5	double closed
Anas platyrhynchos	Cadherin-18	100	91.37	Yes/Yes	EOA99298.1	A0A493U2B5	double closed
Callorhinchus mili	Cadherin-18 (predicted)	100	75.06	Yes/Yes	XP_007899915.1	A0A4W3IDI2	double closed
Aquila chrysaetos chrysaetos	cadherin-18 isoform X2	100	91.39	Yes/Yes	XP_029897709.1	A0A663FAS0	double closed
Gallus gallus	cadherin-18 isoform X4	100	91.77	Yes/Yes	XP_426046.4	A0A1D5PZT9	double closed
Balaenoptera acutorostrata scammoni	cadherin-18 isoform X1	100	96.58	Yes/Yes	XP_007188294.1	A0A384ALT3_BALAS	double closed
Ursus maritimus	cadherin-18 (predicted)	100	96.96	Yes/Yes	XP_008688109.1	A0A384C0Z5	double closed
Ornithorhynchus anatinus	cadherin-18	100	89.57	Yes/Yes	XP_001507828.2	F6VFP8	double closed
Xenopus laevis	cadherin-18-like isoform X2 (predicted)	100	83.8	Yes/Yes	XP_018124907.1	A0A1L8FS65	incomplete closed
Terrapene carolina triunguis	Cadherin-18	100	62.12	Yes/No	XP_029768360.1	A0A674JC42	incomplete closed
Anolis carolinensis	cadherin-18 isoform X1 (predicted)	100	90.76	Yes/Yes	XP_008112423.1	H9GMS7	incomplete closed
Mastacembelus armatus	cadherin-18-like isoform X1	100	75.88	Yes/Yes	XP_026147849.1	A0A3Q3MBN2	incomplete closed
Salmo trutta	cadherin-18-like (uncharacterized)	99	74.24	Yes/Yes	XP_029612053.1	A0A673X3T7	incomplete closed
Takifugu rubripes	cadherin-18	100	73.87	Yes/Yes	XP_003968224.1	A0A674MQU2	incomplete closed
Lates calcarifer	cadherin-18 (predicted)	100	75	Yes/Yes	XP_018534785.1	A0A4W6G877	incomplete closed
Loligo vulgaris	No results						
Octopus vulgaris	protocadherin Fat 1-like isoform X3	67	32.46	No/No	XP_029644443.1	-	closed
Caenorhabditis elegans	protocadherin Fat 1-like isoform X3	67	30.66	No/No	NP_506256.3	G5EDK5	none
Lumbricus terrestris	No results						
Apis mellifera	fat-like cadherin-related tumor suppressor homolog isoform X9	71	33.98	No/No	XP_026296988.1	-	open
Drosophila melanogaster	Cadherin-N, isoform L	83	30.37	No/No	NP_001027277.1	Q4ABE7	open

Supplementary Figure 12. CDH18 protein conservation and cardiovascular development

Phylogenetic analysis of the CDH18 protein sequence in cardiovascular double-circuit, incomplete closed circuit and open circuit species.

Supplementary Figure 13

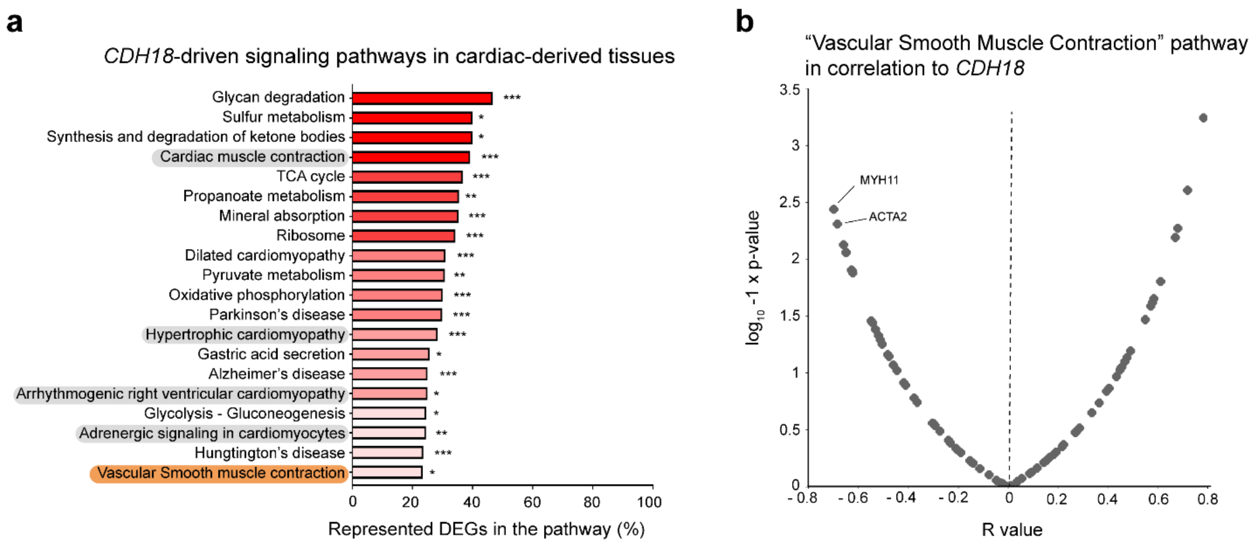

Supplementary Figure 13. CDH18 related signaling pathways

a, KEGG pathway activity correlation pattern analysis for *CDH18* expression in cardiovascular system-derived tissues [GSE7307; aorta, coronary-artery, heart, heart atrium, heart ventricle; total *n*=15; Pearson R; * *p*<0.05, ** *p*<0.01, *** *p*<0.001]. **b**, Volcano plot analysis for genes in cardiovascular system-derived tissues in correlation to *CDH18* [GSE7307; aorta, coronary-artery, heart, heart atrium, heart ventricle; total *n*=15].

Supplementary Figure 14

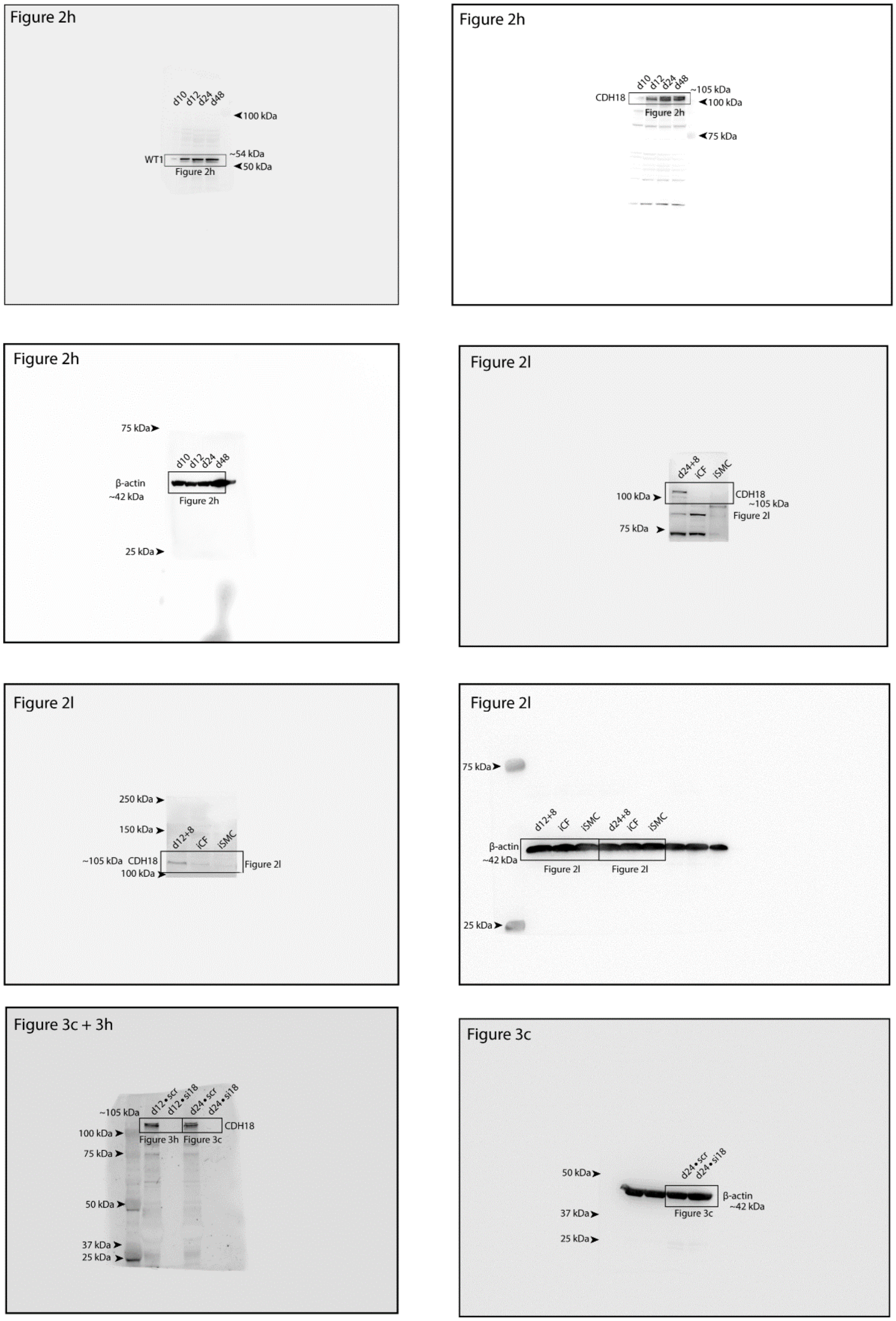

Supplementary Figure 14. Unprocessed blots of Figure 2 and 3

Supplementary Figure 15

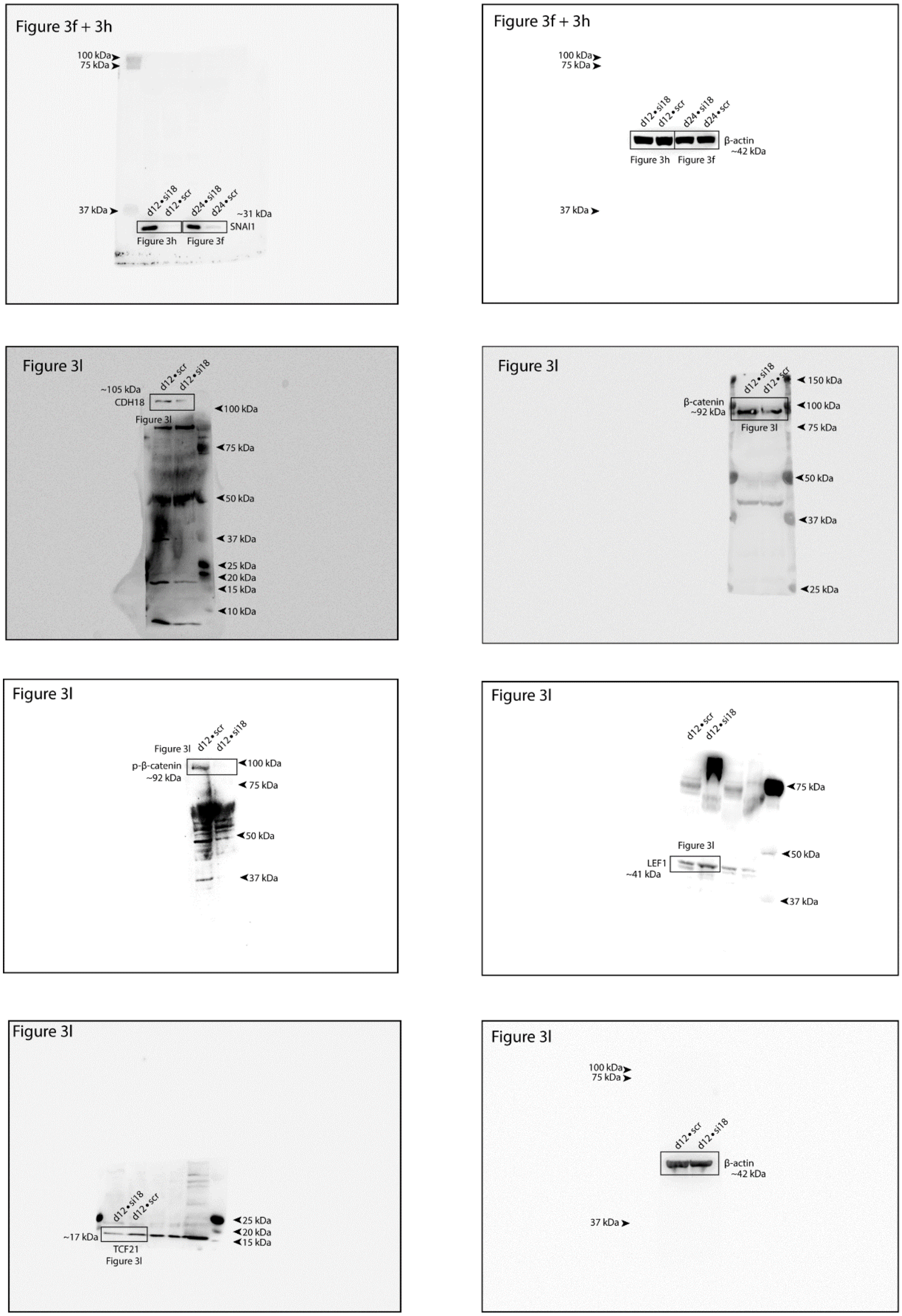

Supplementary Figure 15. Unprocessed blots of Figure 3

Supplementary Figure 16

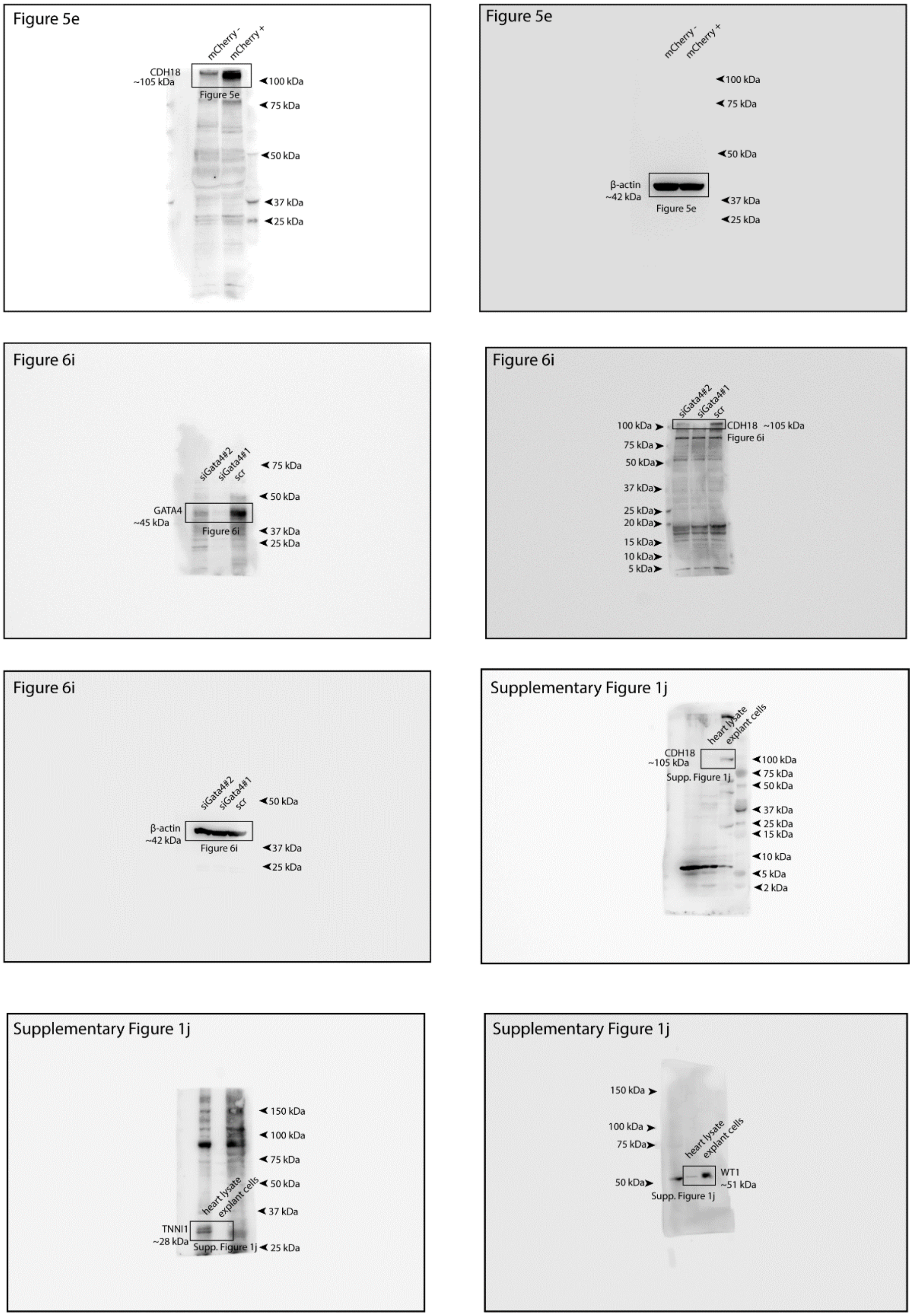

Supplementary Figure 16. Unprocessed blots of Figure 5, 6 and Supplementary 1

Supplementary Figure 17

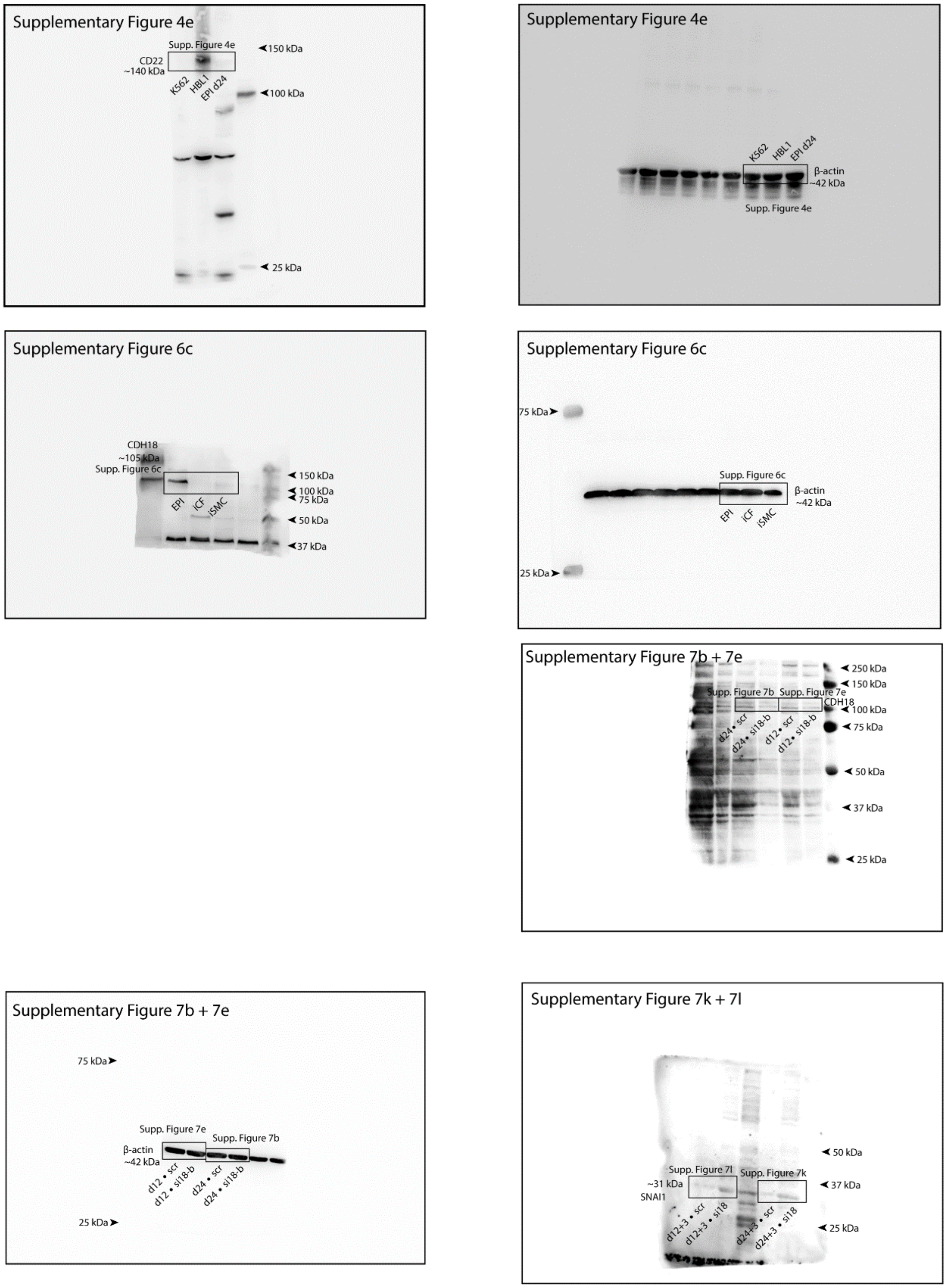

Supplementary Figure 17. Unprocessed blots of Supplementary Figure 4, 6 and 7

Supplementary Figure 18

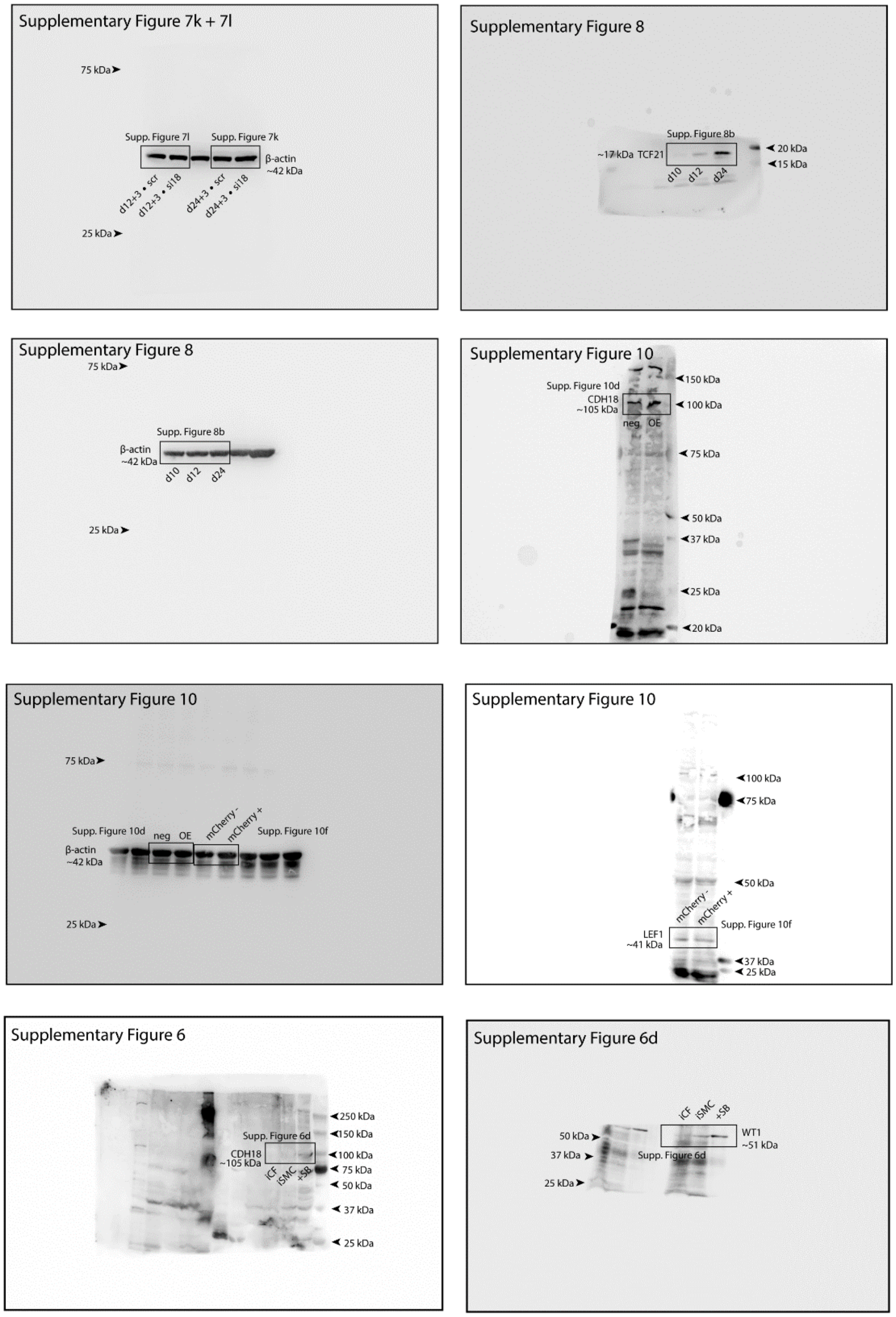

Supplementary Figure 18. Unprocessed blots of Supplementary Figure 7, 8, 10 and 6.

Supplementary Table 1a: Optimization of transfection time

FuGENE®HD: 3µg plasmid/6-well	2:1	3:1	4:1
+24h (ST)	3.95%	2.57%	2.42%
+48h (ST)	**12.50%**	8.00%	7.00%
+48h (RT)	11.70% *		

Supplementary Table 1b: Optimization of transfection agent

Transfection reagent 2:6µg plasmid/6-well	FuGENE®HD	FuGENE®6	PEI	Lipofectamine™ 3000
+48h (ST)	14.60%	18.20%	4.38%	
+72h (ST)	14.70%	**24.60%**		28.80% *
+72h (RT)		13.30%		

Supplementary Table 1. Optimization of transfection protocol

a, b, Optimization of the transfection protocol showing percentage of mCherry+ cells. The left column shows the time after the transfection using either the standard transfection (ST) or reverse transfection (RT) method. **a** 3 µg plasmid DNA per one well of a 6-well-plate and FuGENE®HD transfection reagent was used to assess different transfection agent-to-DNA ratios (upper column). **b** 6 µg plasmid DNA per one well of a 6-well-plate was used to assess different transfection agents (upper column). Albeit transfection efficiency with Lipofectamine 3000 was higher, due to high cell toxicity the total amount of mCherry+ sorted cells was low. [* indicates conditions with low cell survival, bold marked values indicate highest absolute amount of transfected cells]

Supplementary Table 2

Genes	Sequences (5' – 3')
WT1	**F:** ATAGGCCAGGGCATGTGTATGTGT
	**R:** AGTTGCCTGGCAGAACTACATCCT
TBX18	**F:** TTAACCTTGTCCGTCTGCCTGAGT
	**R:** GTAATGGGCTTTGGCCTTTGCACT
ALDH1A2	**F:** TTTGCCAAGTTCCATTGTGCCAGG
	**R:** TGGTGGAGTCACTGGAAAGCAGAA
CD22	**F:** GGTCAGCCTCCAATGTGACT
	**R:** CTGGCTCTGTGTCCTCTTCC
CDH18	**F:** AATGACAATCCACCCGAAC
	**R:** GGCTGTGTTATCTTCATTGTCC
CDH1	**F:** TTCTGCTGCTCTTGCTGTTT
	**R:** TGGCTCAAGTCAAAGTCCTG
CDH2	**F:** CTCCAATCAACTTGCCAGAA
	**R:** ATACCAGTTGGAGGCTGGTC
ACTA2	**F:** TCAATGTCCCAGCCATGTAT
	**R:** CAGCACGATGCCAGTTGT
CNN1	**F:** AAGGACGCACTGAGCAACGCTATT
	**R:** ACGCCACTGTCACATCCACATAGT
GAPDH	**F:** TGATGACATCAAGAAGGTGGTGAAG
	**R:** TCCTTGGAGGCCATGTGGGCCAT
TCF21	**F:** AGGCAGATCCTGGCTAACGACAAA
	**R:** TCCAGGTACCAAACTCCAAGGTCA
GATA4	**F:** TTCCAGCAACTCCAGCAACG<br>**R:** GCTGCTGTGCCCGTAGTGAG

Supplementary Table 2. List of qRT-PCR primers
