# Supplementary material for: CDH18 is a fetal epicardial biomarker regulating differentiation towards vascular smooth muscle cells
Source: NPJ Regen Med. 2022 Feb 2;7:14. doi: 10.1038/s41536-022-00207-w (PMC8810917; doi:10.1038/s41536-022-00207-w)
Supplement: Supplementary file 2 — REPORTING SUMMARY [file 41536_2022_207_MOESM2_ESM.pdf]

nature research

Corresponding author(s): Yoshinori Yoshida and Antonio Lucena-Cacace

Last updated by author(s): Dec 7, 2021

Reporting Summary

Nature Research wishes to improve the reproducibility of the work that we publish. This form provides structure for consistency and transparency in reporting. For further information on Nature Research policies, see our Editorial Policies and the Editorial Policy Checklist.

Statistics

For all statistical analyses, confirm that the following items are present in the figure legend, table legend, main text, or Methods section.

n/a	Confirmed	
☐	☒	The exact sample size (n) for each experimental group/condition, given as a discrete number and unit of measurement
☐	☒	A statement on whether measurements were taken from distinct samples or whether the same sample was measured repeatedly
☐	☒	The statistical test(s) used AND whether they are one- or two-sided<br>*Only common tests should be described solely by name; describe more complex techniques in the Methods section.*
☒	☐	A description of all covariates tested
☐	☒	A description of any assumptions or corrections, such as tests of normality and adjustment for multiple comparisons
☐	☒	A full description of the statistical parameters including central tendency (e.g. means) or other basic estimates (e.g. regression coefficient) AND variation (e.g. standard deviation) or associated estimates of uncertainty (e.g. confidence intervals)
☐	☒	For null hypothesis testing, the test statistic (e.g. F, t, r) with confidence intervals, effect sizes, degrees of freedom and P value noted<br>*Give P values as exact values whenever suitable.*
☒	☐	For Bayesian analysis, information on the choice of priors and Markov chain Monte Carlo settings
☒	☐	For hierarchical and complex designs, identification of the appropriate level for tests and full reporting of outcomes
☐	☒	Estimates of effect sizes (e.g. Cohen's d, Pearson's r), indicating how they were calculated

Our web collection on statistics for biologists contains articles on many of the points above.

Software and code

Policy information about availability of computer code

Data collection	RNA-seq: llumina Nextseq 500<br>Fluoresence/Phase contrast Microscopy: BZ-X710 (Keyence)<br>Flow Cytometry/FACS: BD FACS Aria II Special Order System (BD)<br>Wester Blot: LAS4000 (Cytiva)<br>qPCR: StepOnePlus (AppliedBiosystems)
Data analysis	RNA-seq: R (3.6.1); NOISeq (2.28.0)<br>Flow cytometric analysis: BD FACSDiva (v6 and v8) and FlowJo (v10)<br>Imaging: BZ-X Analyzer (Keyence); ImageJ (v 1.52)<br>qPCR: StepOnePlus (AppliedBiosystems)<br>GraphPad Prism (v7 and higher)

For manuscripts utilizing custom algorithms or software that are central to the research but not yet described in published literature, software must be made available to editors and reviewers. We strongly encourage code deposition in a community repository (e.g. GitHub). See the Nature Research guidelines for submitting code & software for further information.

Data

Policy information about availability of data

All manuscripts must include a data availability statement. This statement should provide the following information, where applicable:

- Accession codes, unique identifiers, or web links for publicly available datasets
- A list of figures that have associated raw data
- A description of any restrictions on data availability

The RNA-seq data reported in this paper have been submitted to NCBI's Gene Expression Omnibus with the accession codeGSE165450. The data that support the findings of this study are available upon reasonable request.

Field-specific reporting

Please select the one below that is the best fit for your research. If you are not sure, read the appropriate sections before making your selection.

☒ Life sciences ☐ Behavioural & social sciences ☐ Ecological, evolutionary & environmental sciences

For a reference copy of the document with all sections, see nature.com/documents/nr-reporting-summary-flat.pdf

Life sciences study design

All studies must disclose on these points even when the disclosure is negative.

Sample size	Required experimental sample sizes were estimated based on previous established protocols in the field. The sample sizes were adequate as the differences between experimental groups were reproducible. All n values are clearly indicated within the figure legends.
Data exclusions	GraphPad Outliner calculator was used to exclude outliners.
Replication	All experiments were repeated multiple times as indicated in each figure legend.
Randomization	Randomization was not carried out in experiments.
Blinding	Cell counting in invasion assay was performed under blinding conditions.

Reporting for specific materials, systems and methods

We require information from authors about some types of materials, experimental systems and methods used in many studies. Here, indicate whether each material, system or method listed is relevant to your study. If you are not sure if a list item applies to your research, read the appropriate section before selecting a response.

Materials & experimental systems

n/a	Involved in the study	
☐	☒	Antibodies
☐	☒	Eukaryotic cell lines
☒	☐	Palaeontology and archaeology
☐	☒	Animals and other organisms
☒	☐	Human research participants
☒	☐	Clinical data
☒	☐	Dual use research of concern

Methods

n/a	Involved in the study	
☒	☐	ChIP-seq
☐	☒	Flow cytometry
☒	☐	MRI-based neuroimaging

Antibodies

Antibodies used

rabbit WT1 (Abcam, ab89901, dilution 1:100-200)
mouse ZO1 (Invitrogen, ZO1-1A12, dilution 1:100 – 1:300)
rabbit POSTN (ThermoFisher, PA5-98301, dilution 1:200)
mouse α-SMA (Abcam, ab7817, dilution 1:200)
mouse Ki67 (BioLegend, 350502, dilution 1:200)
rabbit WT1-Alexa488 (Abcam, ab202635, dilution 1:50-200)
rabbit IgG-Alexa488 (Abcam, ab199091, dilution 1:50)
rabbit CDH18-FITC (Biorbyt ltd, orb7854, dilution 1:50)
mouse CD31-APC (BioLegend, 303116, dilution 1:50)
mouse CD144-FITC (BD, 560874, dilution 1:50)
mouse CD22-APC (BD, 562860, dilution 1:50)
rabbit CDH18 antibody (Proteintech 13091-1-AP, dilution 1:1000)

rabbit CD22 ((Abcam, ab207727, dilution 1:1000)
rabbit GATA4 (D3A3M) (CST, 36966, dilution 1:1000)
rabbit SNAI1 (Abcam, ab63371, dilution 1:1000)
mouse β-actin (Sigma, A5441, dilution 1:1000)
rabbit TCF21 (Abcam, ab32981, dilution 1:1000)
rabbit β-catenin (CST, 8814, dilution 1:1000)
rabbit phospo-β-catenin (S33/S37/T41) (CST, 9561, dilution 1:1000)
rabbit LEF1 (Bethyl Laboratories, A303-486A, dilution 1:1000)
mouse cardiac Troponin I [4C] (Abcam, ab10231, dilution 1:1000)
goat-anti-mouse-Alexa488 (Invitrogen, A11001, dilution 1:1.000-2.000)
goat-anti-rabbit-Alexa546 (Invitrogen, A11010, dilution 1:1.000-2.000)
goat-anti-mouse-Alexa546 (Invitrogen, A11030, dilution 1:1.000-2.000)
goat-anti-mouse-Alexa594 (Invitrogen, A11032, dilution 1:1.000-2.000)
goat-anti-rabbit-Alexa647 (Invitrogen, A21245, dilution 1:1.000-2.000)
goat-anti-mouse-Alexa647 (Invitrogen, A21236, dilution 1:1.000-2.000)
goat anti-rabbit-horseradish peroxidase (Abcam, ab97051, dilution 1:5000)
rabbit anti-mouse-horseradish peroxidase (Abcam, ab97046, dilution 1:5000)

Validation: Antibodies were validated by the manufacturer, previous publications and/or with positive and negative control samples.

Eukaryotic cell lines

Policy information about cell lines

Cell line source(s): The 201B7, 409B2 and MYH6-EIP4 hiPSC lines used in this study were generated in CiRA. MEC1 cell line was obtained from Merck (SCC187). Commercially purchased HBL1 and K562 cell lines were provided.

Authentication: All hiPSC used in this study including reporter lines were validated with karyotype testing.

Mycoplasma contamination: All cell lines are regularly tested and were always negative for mycoplasma

Commonly misidentified lines (See ICLAC register): No commonly misidentified lines were used in this study.

Animals and other organisms

Policy information about studies involving animals; ARRIVE guidelines recommended for reporting animal research

Laboratory animals: In this study C57BL/6NCrSlc female pregnant mouse with E14 embryos were sacrificed to obtain mouse fetal heart at E14.

Wild animals: We did not use any wild animal in this work.

Field-collected samples: We did not use field-collected samples in this work.

Ethics oversight: All experimental protocols involving animals were approved by the Kyoto University Animal Experimentation Committee, and procedures were performed in accordance with the Guidelines for Animal Experiments of Kyoto University and the Guide for the Care and Use of Laboratory Animals by the Institute of Animal Resources.

Note that full information on the approval of the study protocol must also be provided in the manuscript.

Flow Cytometry

Plots

Confirm that:

☒ The axis labels state the marker and fluorochrome used (e.g. CD4-FITC).

☒ The axis scales are clearly visible. Include numbers along axes only for bottom left plot of group (a 'group' is an analysis of identical markers).

☒ All plots are contour plots with outliers or pseudocolor plots.

☒ A numerical value for number of cells or percentage (with statistics) is provided.

Methodology

Sample preparation: Sample preparation was carried out as detailed in the Online Methods.

Instrument: BDFACS AriaII Special Order System

Software: Acquisition: BD FACSDiva (v6 and v8)
Analysis : Flowjo (v10)

Cell population abundance	Flow cytometry analysis populations were generally >15%.
Gating strategy	Cells were gated on FSC/SSC first and an unstained sample and IgG isotype as controls to establish the gate for positive cells. Regarding mCherry-positive gate, cells were gated on FSC/SSC first and a empty non-plasmid containing transfected sample as control to establish the gate for positive cells.

☒ Tick this box to confirm that a figure exemplifying the gating strategy is provided in the Supplementary Information.
